# Spatio-temporal characteristics of Tuberculosis in Ghana

**DOI:** 10.12688/f1000research.109053.1

**Published:** 2022-02-16

**Authors:** Abdul-Karim Iddrisu, Emmanuel A. Amikiya, Francis Kwame Bukari

**Affiliations:** 1 Mathematics and Staistics, University of Energy and Natural Resources, Sunyani, Brong Ahafo, +233, Ghana; 2 Department of Management Science, Ghana Institute of Management and Public Administration, Accra, Greater Accra, +233, Ghana

**Keywords:** Bayesian spatial and space-time models, Tuberculosis relative risk, baseline predictors and TB hot-spots.

## Abstract

Background: The number of Tuberculosis (TB) cases or deaths is declining, however, the rate of decline is not adequate to meet the World Health Organization's (WHO's) mitigation. TB remains a public health problem in Ghana with a significant economic and health burden on its citizens and health care system. Consequently, there is a need for further studies about the disease aimed at accelerating the rate of decline in cases.

Methods: The spatio-temporal characteristics of TB in Ghana using Bayesian spatial and spatio-temporal regression models was analysed in this study. Data were obtained from Ghana National Tuberculosis Programme (NTP) for the 10 regions of Ghana, collected over a six-year period. The study also examines some baseline predictors of TB infections to ascertain their effects on the TB risk across the ten regions in Ghana.

Results: Hot-spots of TB cases are observed in the Upper East, Upper West, Volta, Western, and Central regions and low risk in the Northern, Ashanti, Greater Accra, Brong Ahafo, Eastern and Western regions. The results indicated a clustering of risk between neighboring regions. TB cure rate, TB success rate, knowledge about TB, awareness that TB is airborne, HIV prevalence, percentage of literacy, and high income are important predictors of detection for this disease across the ten regions of Ghana.

Conclusion: Most regions in Ghana have similar TB risks. A substantial reduction in TB cases requires measures that will increase detection, success and cure rates, awareness, knowledge about how this disease spreads as well adequate health facilities with easy access.

## Background

Globally, Tuberculosis (TB) is in the top ten causes of death in low-income countries (ranking above HIV/AIDS). ^1^ This infectious disease is transmitted by *bacillus Mycobacterium tuberculosis.* ^2^ TB occurs essentially in individuals with weakened immune systems than those with healthy immune systems. As HIV weakens the immune system, TB is often seen in individuals with HIV. ^3^ Nevertheless, in 2019, approximately 10 million people tested positive for TB with an estimated 1.2 million HIV-negative deaths. ^2^ Although this disease can affect both sexes, reports have shown that males (aged 15 years and above) accounted for 56% of the global infections compared to 32% for females in the same age group. Infections among children (aged 15 years and below) accounted for 12% of the total cases and about 8.2% of the reported cases were HIV-patients. ^2^ Geographically, the 2020 reports have shown that the highest number of new TB cases occurred in the WHO South-East Asia (43%), followed by Africa Region (25%), and the WHO Western Pacific (18%), compared to Eastern Mediterranean (8.2%), America (2.9%) and Europe (2.5%). ^2^^,^^4^ It has been estimated that 86% of new TB cases occurred in the 30 high TB burden countries, ^4^ where eight countries including India, China, Indonesia, Philippines, Pakistan, Nigeria, Bangladesh and South Africa accounted for 2/3 of the new TB cases.

As such, in order to have faster reduction in TB incidents and deaths worldwide and especially in low-income countries, the World Health Organization (WHO) has called for the development of TB vaccines. ^2^ Reports have shown that TB is preventable and can also be cured as well. Studies have reported that approximately 85% of those who develop TB disease can be treated successfully with a 6-month drug regimen. ^2^ Available reports indicate that this treatment has prevented more than 60 millions deaths from 2000 to 2020. ^2^ Despite this treatment, the global and in specific WHO regions and many high TB burden countries did not have a fast enough progression towards the 2020 milestone of The End TB Strategy. The global cumulative reduction was reported at 9% between 2015 and 2019, and about 2.3% between 2018 and 2019. ^2^ However, more optimistically, Europe had achieved 19% reduction in cases and 31% reduction in deaths between 2015 and 2019. Africa had achieved a 16% reduction in cases and a 19% reduction in deaths between 2015 and 2020. ^2^ As the WHO’s target for global decline of TB cases and deaths (2015-2020) were not achieved, there is still a need for further studies to be conducted on the dynamics of the disease and on mitigation measures for TB in Africa. Ghana as a developing country in Africa, has been affected by the respiratory disease and currently has challenges in eradicating TB. The country implemented policies called Directly Observe Therapy (DOT) and National Tuberculosis Programmes (NTPs) in 1994, to detect and treat TB. ^2^^,^^5^^–^^7^ The implementation of the NTP led to 100% DOTs coverage in 2005 with more TB cases detected for treatment every year since. For instance, TB cases detected increased from 7,425 in 1996 to 15,286 in 2009. ^8^^,^^9^

Although TB cases and deaths have declined due to the implementation of mitigation/treatment strategies, TB still remains a life-threatening disease and poses a burden on health infrastructure in Ghana. Hence, TB has gained considerable attention as a topic of research among researchers from diverse backgrounds. Studies have investigated the dynamics of TB indicators as well as risk factors of this disease in Ghana ^6^^,^^7^^,^^10^ Osei *et al.* ^6^^,^^7^ studied trends of TB detection and treatment outcomes using the logistic regression to assess the relationship between patients and disease characteristics. Further, Osei *et al.* ^6^^,^^7^ have studied TB detection, mortality and co-infection with HIV, using patients data collected in the Volta Region from 2012–2016. The authors used simple and multiple logistic regression to investigate determinants of TB mortality in 10 districts of the Volta Region of Ghana. Aryee *et al*. ^10^ have studied the dynamics of TB using Autoregressive Moving Average (ARIMA) methods and TB data recorded by the Korle Bu Teaching Hospital from 2008–2017. Iddrisu *et al.* ^11^ have studied the temporal and geographical pattern of TB prevalence in Ghana between 2015 and 2018.

In this paper, Bayesian hierarchical spatial and space-time models is used to study the relative risk (RR) of TB and associated risk factors across the 10 Regions of Ghana. Hence, the purpose of this study was to model the spatio-temporal risk pattern of TB in Ghana, using Bayesian hierarchical and space-time models discussed in previous literature. ^12^^–^^16^

## Methods

### TB cases

In this study TB detection data obtained from Ghana Health Service and National Tuberculosis Programme was used. ^8^ The data contained information on TB detection from 2009 to 2017, for the 10 old administrative regions of Ghana. These regions include, Ashanti, Brong Ahafo, Central, Eastern, Greater Accra, Northern, Upper East, Upper West, Volta, and Western.

**Figure 1.**
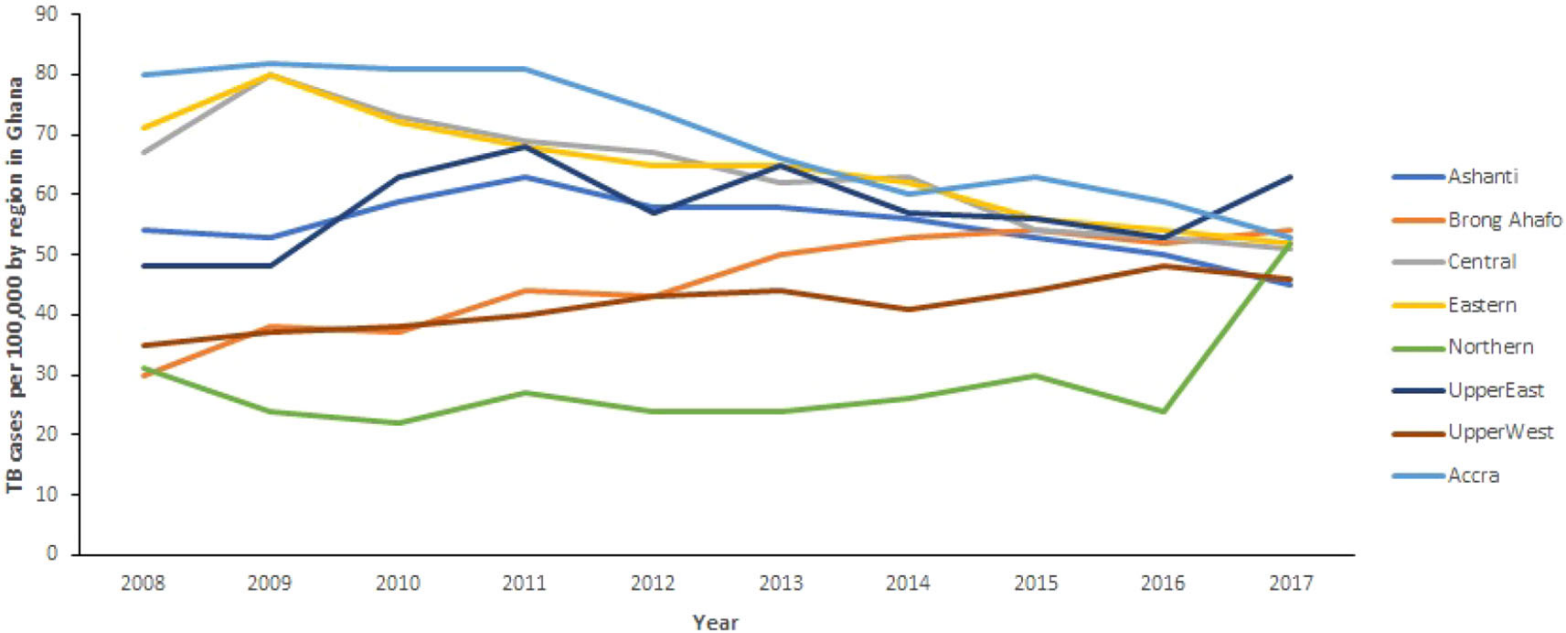
Trend of TB cases detection per 100,000 by region for 10 years from 2008 to 2017 in Ghana.

Figure 1 shows the TB trends in the 10 regions from 2008 to 2017. Generally, there is a decrease in TB cases observed in all regions (except Brong Ahafo Region where TB cases increase) of Ghana from 2008 to 2016. It can also be observed that TB cases in Northern and Upper East increased remarkably between 2016 and 2017, whereas cases in Ashanti Region decreased from 50 per 100, 000 population in 2016 to 45 per 100,000 population in 2017. In the Northern Region, TB cases increased from 24 per 100,000 population in 2016 to 52 per 100,000 population in 2017. Further, cases in Upper East Region increased from 53 per 100,000 population in 2016 to 63 per 100,000 population in 2017. However, the changes in TB cases in the other regions are almost horizontal.

**Figure 2.**
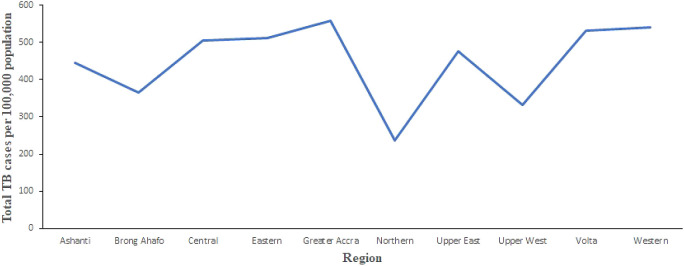
Trend of the total TB cases detection per 100,000 for each region from 2008 to 2017 in Ghana.

**Figure 3.**
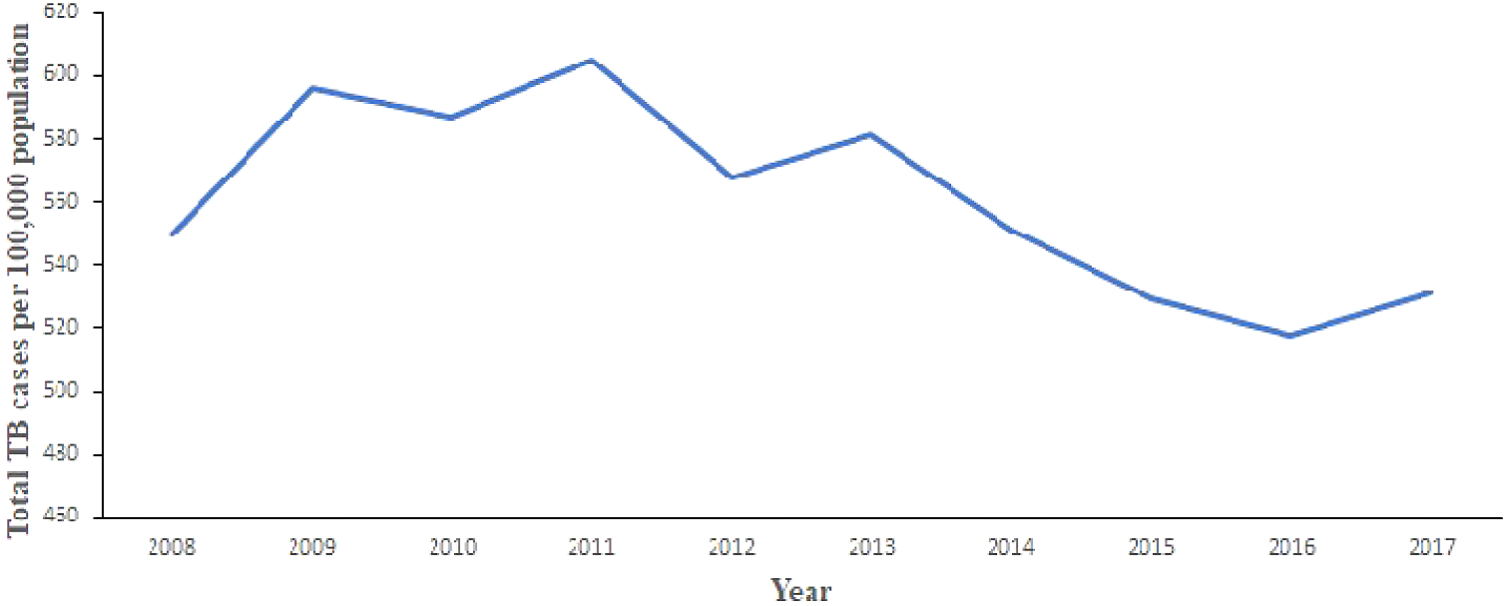
Trend of the total TB cases detection per 100,000 population for each year.

Figure 2 shows the trend of total number of TB cases for each region from 2008 to 2017. It shows that the highest cases recorded was in the Greater Accra Region from 2008 to 2017. It also shows that the lowest was recorded in the Northern Region. Volta and Western Regions are second and third, respectively, with records slightly lower than Greater Accra Region. In addition, Figure 3 shows the trend of total TB cases in each year/period. The figure shows that the highest number of total TB cases was recorded in 2011 while the lowest was in 2016. It can be observed that TB cases decreased slowly from 2011 to 2017 with increments in 2012 and 2017.

**Figure 4.**
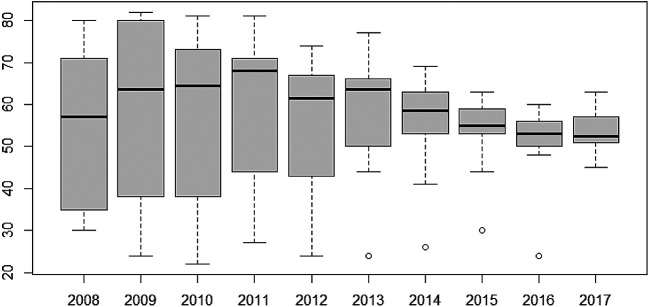
Box-and-whisker plot of TB cases detection per 100,000 population for each year.

**Figure 5.**
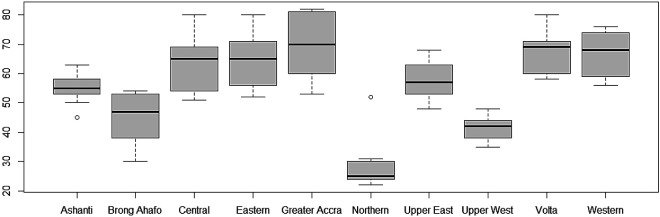
Box-and-whisker plot of TB cases detection per 100,000 population by the 10 old administrative Regions in Ghana.

Furthermore, the variability of TB cases from 2008 to 2017 have also been presented using box-and-whisker plots in Figure 4. The overlapping box-and-whisker plots imply that there is no variability in cases among the years. The plots show that TB cases were skewed towards larger numbers from the year 2008 to 2014, and skewed towards smaller numbers from 2015 to 2016. Extremely small numbers were observed in 2013, 2014, 2015, and 2017. Variability across the regions have been presented in Figure 5. None-overlap of box-and-whisker plots imply variability between regions. Thus, there is variability in TB cases among the regions since some of the box-and-whisker plots do not overlap. It can also be observed that TB cases in most of the regions are skewed towards larger numbers except Northern and Upper East Regions (especially, the Northern Region with one extremely large value).

### Baseline predictors

Some baseline regional characteristics on the risk of TB infection have been explored in this study. The set of baseline predictors include doctor to population ratio, nurse to population ratio, HIV prevalence, TB cure rate, TB success rate, wealth quantiles and the proportions of men/women employed, unemployed, educated, and uneducated. Variables also considered in the study include proportions of people who have heard about the TB disease, have knowledge that TB is airborne, knowledge that TB can be cured, and those who believe that TB status should be kept secret. In the data analyses, all the baseline variables were explored to obtain significant predictors of the TB cases.

### Hierarchical space model

For spatial TB data, let $y_{i},i=1,\ldots ,n$ denote a Poisson random variable with probability mass function defined as $P\left(y_{i}|\theta \right)$ , where $\theta =\left(\theta_{1},\ldots ,\theta_{n}\right)$ is a vector of relative risk parameters for each region. The variable $y_{i}$ represent total number of TB cases for region i. It follows that the likelihood function for the Poisson variable $y_{i}$ is defined as:

$$P\left(y|\theta \right)=\prod_{i=1}^{n}P\left(y_{i}|\theta \right),$$

with assumption that the sample values of $y={\left(y_{1},\ldots ,y_{n}\right)}^{\prime }$ given the parameter estimates θ are independent. ^17^ Bayesian modeling framework requires prior distribution of the unknown parameters in the likelihood function for the data. The prior distribution represents the current knowledge of the parameters θ before the data $y_{i}$ are observed. ^17^ Under the Bayesian framework, all parameters are stochastic and assigned appropriate distributions called prior distributions. ^17^ Bayesian modeling framework combines the likelihood function for the data and the prior distributions for the parameters resulting in a distribution known as the posterior distribution. ^15^^,^^17^^–^^19^ The posterior distribution is defined as $P\left(\theta |y\right),$ (i.e. probability distribution of the parameters given that the data which is proportional to the product of the likelihood function) while the prior distribution is defined as:

$$P\left(\theta |y\right)=\frac{P\left(y|\theta \right)P\left(\theta \right)}{\int_{p}L\left(y|\theta \right)P\left(\theta \right)d_{\theta }}$$ (1)

where the denominator of Equation (1) is called the normalizing constant. It has been shown that the posterior distribution can alternatively be written as:

$$P\left(\theta |y\right)\propto P\left(y|\theta \right)P\left(\theta \right),$$

Moreover, after covariate adjustments, statistical inferences remained unchanged and the classical parametric formulation (in Equation 7) remains the most accurate model for the TB data. The posterior summary statistics in Table 6 showed negligible risk of TB across the 10 regions. Precisions of both the unstructured and structured components indicate clustering of risk. The models are implemented in R-software via the Integrated Nested Laplace Approach (INLA) packagon. Instructions on downloading and how to use this package can be found in Håvard *et al*. ^20^ Also, this package can directly be installed in R software by using the command install.packages("INLA",repos=c (getOption("repos"),INLA=" https://inla.r-inla-download.org/R/stable "), dep=TRUE).

### Correlated and uncorrelated heterogeneity structures

The clustering and variability of risk were studied using correlated and uncorrelated structures, respectively. The use of uncorrelated heterogeneity models with gamma or beta prior distributions for estimating the relative risk of a given disease are useful, however, such models have limitations. Andrew [ 17, P. 82-84] stated that a gamma distributions restrict the incorporation of covariates into the modeling process. Another limitation is that such models do not allow the formulation of a simple and adaptable general form of the gamma distribution with spatially correlated parameters [ 17, P. 82-84]. Wolpert and Ickstadt ^21^ have also given an example of correlated gamma field models that yield poor results. ^22^ However, Gaussian models permit incorporation of correlated structure (CH) into the modeling process. Further, variability in the data can be modeled as uncorrelated heterogeneity (UH) using a Gaussian prior distribution with a mean zero and risk variance of the disease across the regions. Both correlated and uncorrelated heterogeneity can be incorporated into the model to account for clustering and heterogeneity of risk. These structures are introduced into the modeling through a log-linear term with additive random effects. ^18^^,^^23^ Besag *et al*. ^24^ have provided the form of the model with CH and UH structures parameterized as follows:

$$\exp\left(x_{i}^{\prime }\beta +\phi_{i}+u_{i}\right),$$

where $x_{i}^{\prime }\beta$ is the fixed effect component, $\phi_{i}$ and $u_{i}$ are the correlated and uncorrelated heterogeneity components, respectively with separate prior distributions. Often, the CH component is assumed to have either an intrinsic Gaussian conditional auto-regressive (CAR) prior distribution or a fully specified Multivariate Normal prior distribution. ^18^^,^^19^^,^^22^^,^^24^

### Conditional Auto-regressive (CAR) models

CAR models provide a tool for detecting and identifying regions where disease risks are clustered. The specification of CAR models provide a framework for borrowing strength between neighboring regions in such a way that, regions that share boundaries are likely to have similar risks and regions that are distant apart are likely to show variability with regard to risk. Waldo Tobler’s ^25^ noted that “ *everything is related to everything else but near-by things are more related than distant things*”. CAR models were rarely used to detect and cluster risk until the 1990s. ^13^^,^^26^ The models enable the influence of disease risk in neighboring regions to be modeled and estimated. ^18^^,^^19^^,^^27^ Distances or boundaries between the regions are used to determine neighborhood properties in the CAR models. ^14^^,^^17^^,^^28^

Let $\Omega =\left\{1,2,\ldots ,n\right\}$ denote the study area and $N_{i}=\left\{j\in \Omega :i\in j\right\}$ classifies regions that share boundaries with region i. Let $\phi_{i},i\in \Omega$ be a stochastic variable, then the CH structure of $\phi_{i}$ follows a normal distribution defined as:

$$\phi_{i}|\phi_{j\neq i}∼N\left(\sum_{i\neq j}W_{\mathrm{ij}}\phi_{j},\tau_{i}^{2}\right)$$ (2)

where $W_{\mathrm{ij}}$ is a spatial dependence parameter for quantifying the weight of each observation on the CAR structure $\phi_{i},$ $\tau_{i}^{2}$ is the variance of $\phi_{i},$ and $\phi_{j}$ is a set of all observation except $\phi_{i}.$ The spatial dependence parameter $W_{\mathrm{ij}}$ is non-zero if j∈S, but set to zero if i=j, in order to prevent auto-correlation. ^14^^,^^18^^,^^22^^,^^24^^,^^29^ It can be observed from Model 2 that the $\phi_{i}$ depends only on a set of neighbors $\phi_{j}$ provided the location j is in the neighborhood $N_{i}$ of $\phi_{i}.$

Assume that region i has M neighbors and $W_{\mathrm{ij}}=\frac{1}{M}$ for each region that is a neighbor but zero elsewhere. The conditional expectation of $\phi_{i}$ is given by:

$$E\left[\phi_{i}|\phi_{j\neq i}\right]=\mu_{i}+\sum_{j\in N_{i}}\Phi_{\mathrm{ij}}\left[\phi_{j}-\mu_{i}\right]$$

and the conditional variance is:

$$\mathrm{var}\left(\phi_{i}|\phi_{j}\right)=\tau_{i}^{2}.$$

The Gaussian processes are defined by mean and covariance functions. ^30^ Thus, the mean and variance-covariance functions are required to specify the CAR model. It follows the conditional probability distribution of the CAR structure $\phi_{i}$ is defined as ^22^^,^^24^^,^^25^:

$$f\left(\phi_{i}|\phi_{j\neq i}\in \Omega \right)=\sqrt{\frac{1}{2{\pi \tau }_{i}^{2}}}\exp\left\{-\frac{{\left[\left(\phi_{i}-\mu_{i}\right)-\rho \sum_{j\in N_{t}}W_{\mathrm{ij}}\left(\phi_{j}-\mu_{j}\right)\right]}^{2}}{2\tau_{i}^{2}}\right\},$$ (3)

where $\mu_{i}\in R,\tau_{i}^{2}\in R^{+},|\rho |<1,W_{\mathrm{ij}}\in R,W_{\mathrm{ij}}=W_{\mathrm{ji}},W_{\mathrm{ii}}=0.$ The CAR conditional probability distribution function can be written as ^18^^,^^22^^,^^24^:

$$f\left(\phi_{i}|\phi_{j\neq i}\right)=\frac{1}{{\left(2\pi \right)}^{n/2}\det{\left(B^{-1}\Sigma \right)}^{1/2}}\exp\left[-\frac{{\left(\phi -\mu \right)}^{\prime }\Sigma^{-1}B\left(\phi -\mu \right)}{2}\right],$$

where $\mu \in R^{n}$ is an n− dimensional vector with components $\mu ={\left(\mu_{1},\mu_{2},\ldots ,\mu_{n}\right)}^{\prime },$ $\Sigma \in R^{+\left(n\times n\right)}$ is a symmetric diagonal matrix with components:

$$\Sigma =\mathrm{diag}\left(\tau_{1}^{2},\ldots ,\tau_{n}^{2}\right)$$

and $B\in R^{n\times n}$ is an invertible matrix defined as:

$$B=\left(I-\mathrm{\rho W}\right)\;\text{with}\;B_{\mathrm{ij}}=\left\{\begin{array}{l} 1\;i=j,\\ -\rho W_{\mathrm{ij}}\;j\in N_{i},\\ 0\;\text{otherwise}. \end{array}\right.$$

The symmetry of Σ implies that the covariance matrix $\Sigma^{-1}\;B=B^{-1}\Sigma$ is symmetric such that $W_{\left(\mathrm{ij}\right)}\tau_{j}^{2}=W_{\left(\mathrm{ji}\right)}\tau_{i}^{2},i,j\in S.$ The probability distribution Function (3) can alternatively be defined as:

$$\phi_{i}|\phi_{j\neq i}∼N\left[\mu_{i}+\rho \sum_{j\in N_{i}}W_{\left(\mathrm{ij}\right)}\left(\phi_{j}-\mu_{j}\right),\tau_{i}^{2}\right],i\in \Omega \;\text{and}\;\phi ∼N\left(\mu ,B^{-1}\Sigma \right)$$ (4)

It has been proved that the CAR structure $\phi_{i}$ follows the Gaussian distribution by showing that Σ is symmetric, see ^18^^,^^31^ for details.

*0.0.1. Parameter estimation: CAR model*

Parameters in the CAR model are estimated using Bayesian hierarchical methods. The TB detection data used in the study are counts (whole numbers), therefore, Poisson distribution is assumed for such data. The unknown risk of TB in any region i represented by $\phi_{i}.$ The number of cases and population risk in any region i are denoted by $y_{i}$ and $N_{i},$ respectively. The expected number of cases in region i can then be written as:

$$E_{i}={\mathrm{rN}}_{i},$$

where

$$r=\frac{\sum_{t=1}^{n}y_{i}}{\sum_{i=1}^{n}N_{i}}$$

gescrepresents the overall risk in the study population. The corresponding likelihood function is defined as:

$$\ell \left(\theta_{i}\right)=\prod_{i=1}^{n}\frac{\exp\left(-E_{i}\theta_{i}\right){\left(E_{i}\theta_{i}\right)}^{y_{t}}}{y_{i}!}=P\left(y,E|\theta \right).$$

Taking natural logarithm of the likelihood function, differentiating with respect to the disease risk $\theta_{i}$ and equating to zero, it can be shown that the maximum likelihood estimator ${\hat{\theta }}_{i}$ of $\theta_{i}$ is

$${\hat{\theta }}_{i}=\frac{y_{i}}{E_{i}},$$

which defines the standardized mortality ratio (SMR) in region i. However, using the Bayesian framework, $y_{i}∼$ Poisson $\left(E_{i}\theta_{i}\right)$ , where the Poisson mean $\mu_{i}=$ $E_{i}\theta_{i},\;$ $\theta_{i}∼$ Gamma $\left(\alpha ,\gamma \right)$ with shape parameter α and scale parameters γ, respectively. However, these formulations do not incorporate covariates in the modeling process. Covariates through a linear predictor was introduced, as seen in previous work [ 18, 19, 17, P. 84]. The distribution of the response variable is specified by the exponent of the linear predictor as $y_{i}∼$ Poisson $\left(E_{i}\exp\left(\eta_{i}\right)\right)$ , where $\mu_{i}=E_{i}\exp\left(\eta_{i}\right)$ is the mean of Poisson distribution. Thus, the relative risk of the disease in region i is defined as:

$$\theta_{i}=\exp\left(\eta_{i}\right),$$

where $\eta_{i}=X^{\prime }\beta +\phi_{i},$ and $\phi_{i}$ has a CAR structure.

Using the generalized linear model with a log-link function, we have:

$$\log\left(\mu_{i}\right)=\log\left(E_{i}\right)+X^{\prime }\beta +\phi_{i}.$$

Bayesian models are defined by the posterior distribution of the D parameter estimates, where the posterior is the product of the data likelihood function and the prior distribution(s) of the parameter estimates. Hence, we define the likelihood function as:

$$\ell \left(\beta ,\phi \right)=\prod_{i=1}^{n}\frac{{\left(E_{i}\exp\left(\eta_{i}\right)\right)}^{y_{i}}\exp\left(-E_{i}\exp\left(\eta_{i}\right)\right)}{y_{i}!}=P\left(y,E,\theta |\beta ,\phi \right).$$

The β parameter estimates are assumed to follow the Gaussian distribution defined:

$$P\left(\beta \right)={\left(\frac{1}{2\pi }\right)}^{P/2}{\left(\frac{1}{\tau_{\beta }}\right)}^{P}\exp\left(-\frac{1}{2}\sum_{p=0}^{P}\frac{\beta_{p}^{2}}{\tau_{\beta }^{2}}\right),$$

and the prior distribution for the CAR random effect is defined by:

$$P\left(\phi \right)=\left[\phi_{i}|\phi_{j\neq i},\tau_{\phi }^{2}\right]∼N\left(\sum_{j\neq i}\frac{w_{\mathrm{ij}}}{w_{\mathrm{ij}}^{+}}\phi_{j},\frac{\tau_{\phi }^{2}}{w_{\mathrm{ij}}^{+}}\right)∼\mathrm{CAR}\left(0,\tau_{\phi }^{2}\right)$$

where $w_{\mathrm{ij}}^{+}$ is the number of areas which share boundaries with the $i^{\mathrm{th}}$ area ^32^ with:

$$w_{\mathrm{ij}}=\left\{\begin{array}{ll} 1 & j=i\\ \varphi \left(i,j\right) & j\in N_{i}:\forall i,j\in S,w_{\mathrm{ij}}=w_{\mathrm{ji}}\\ 0 & \text{otherwise} \end{array}\right.$$

with $\varphi \left(i,j\right)$ quantifying the proximity between regions i and j . That is, if $\varphi \left(i,j\right)=1$ , then i and j share a common boundary. The posterior distribution can be expressed as follows:

$$P\left(\beta ,\phi ,\tau_{\beta }^{2},\tau_{\phi }^{2}|y,E,\theta \right)\propto P\left(X,E,\theta |\beta ,\phi ,\tau_{\beta }^{2},\tau_{\phi }^{2}\right)P\left(\beta \right)P\left(\phi \right).$$

The hyperprior distribution for the precision parameters $\tau_{\phi }^{2}$ and $\tau_{\beta }^{2}$ are respectively, $\tau_{\phi }^{2}∼$ Gamma $\left(0.05,0.005\right)$ and $\tau_{\beta }^{2}∼$ Gamma $\left(0.5,0.05\right).$ The linear regression coefficient distribution is defined by:

$$\beta ∼N\left(0,\tau_{\beta }^{2}\right).$$

*0.0.2 The Besag, York and Molli’e (BYM) Model*

Clayton and Kaldor ^33^ were first to propose the BYM framework, and later, Besag *et al*. developed it further. ^24^ The BYM (also known as the convolution model) unifies the CH and UH structures into the same model that is capable of explaining clustering and variability of the disease risk. Although various models have been proposed for smooth risks estimation, the model proposed by Besag *et al.* (BYM) ^24^ have been used extensively in literature. The BYM model is expressed as follows:

$$\eta_{i}=\mu_{i}+\phi_{i}+u_{i}.$$

As indicated in the previous section, the TB cases follow the Poisson distribution, thus, $y_{i}∼$ Poisson $\left(E_{i}\exp\left(\eta_{i}\right)\right),$ where $\mu_{i}=E_{i}\exp\left(\eta_{i}\right).$ The linear link function is $\eta_{i}=X^{\prime }\beta +\phi_{i}+u_{i}.$ The log relative risk is $\log\left(\theta_{i}\right)=\eta_{i}$ . Therefore, the relative risk for area i is defined by:

$$\theta_{i}=\exp\left(X^{\prime }\beta +\phi_{i}+u_{i}\right).$$

The log log-link function is defined as:

$$\log\left(\mu_{i}\right)=\log\left(E_{i}\right)+\left(X^{\prime }\beta +\phi_{i}+u_{i}\right),=\log\left(E_{i}\right)+\log\left(\theta_{i}\right)=\log\left(E_{i}\theta_{i}\right),$$ (5)

where y,β,E and θ are vectors of responses, parameter estimates, the expected number of TB cases and the relative risk of TB, respectively. The $u_{i}$ is the region-specific random effect quantifying the variability of relative risk of the disease.

### Parameter estimation: BYM

Parameters in the BYM are estimated using the same formulations discussed in the previous section, however, $u_{i}$ is required to be a Gaussian prior distribution given by:

$$P\left(u\right)={\left(\frac{1}{2\pi }\right)}^{n/2}{\left(\frac{1}{\tau_{u}}\right)}^{n}\exp\left(-\sum_{i=1}^{n}\frac{u_{i}^{2}}{2\tau_{u}^{2}}\right).$$

The resulting posterior distribution can be written as follows:

$$P\left(\beta ,u,\phi ,\tau_{\beta }^{2},\tau_{u}^{2},\tau_{\phi }^{2}|y,E,\theta \right)\propto P\left(y,E,\theta |\beta ,u,\phi ,\tau_{\beta }^{2},\tau_{u}^{2},\tau_{\phi }^{2}\right)P\left(\beta \right)P\left(u\right)P\left(\phi \right).$$

The distributions for the hyper-prior precision parameters are as follows:

$$\tau_{u}^{2}∼\text{Gamma}\left(0.5,0.005\right),\tau_{\phi }^{2}∼\text{Gamma}\left(0.5,0.005\right)\;\text{and}\;\tau_{\beta }^{2}∼\text{Gamma}\left(0.5,0.01\right),$$

respectively. The regression coefficients β follow Gaussian distributions stated as follows;

$$\beta ∼N\left(0,\tau_{\beta }^{2}\right).$$

The estimates, $\tau_{u}^{2}$ and $\tau_{\phi }^{2}$ are precision-variance estimates for u and ϕ, respectively, and are used to measure the level of variability of risk among the regions and to cluster risk between neighboring regions. ^18^^,^^27^

### Space-time models

The TB data used in study are collected over time and hence spatial models alone will not be enough to model the space-time pattern of the relative risk of the disease. The spatial models are constrained for identifying heterogeneity and clustering of risk at a single time point. Several methods have been proposed to account for spatial and temporal patterns of disease risks. ^23^^,^^24^^,^^34^^–^^36^

In this section, space-time models are presented based on three modeling frameworks developed by Knorr-Held *et al.,* ^16^^,^^37^ Bernardineli *et al.* ^23^ and Waller *et al.* ^37^ These models differ with regards to their space-time interactive structures and inclusion of covariates. Consider region i, in year t, that recorded $y_{\mathrm{it}}$ TB cases. The cases follow the Poisson distribution, i.e.:

$$y_{\mathrm{it}}∼\text{Poisson}\left(E_{\mathrm{it}}\exp\left(\eta_{\mathrm{it}}\right)\right),$$

where the unknown relative risk at region i in time t is:

$$\theta_{\mathrm{it}}=\exp\left(\eta_{\mathrm{it}}\right),$$

and $E_{\mathrm{it}}$ is the expected number of TB cases in region i in time t . The expected number of TB cases represents the number of cases expected if the population of region i has statistical behavior comparable to the standard population $N_{\mathrm{it}}^{s}.$ We express the crude rate of TB cases for region i in time t as:

$$r_{\mathrm{it}}^{s}=\frac{y_{\mathrm{it}}^{s}}{N_{i}^{s}},$$

and the number of TB cases expected in region i in time t, as:

$$E_{\mathrm{it}}=r_{\mathrm{it}}^{s}N_{\mathrm{it}}=\frac{y_{\mathrm{it}}^{s}}{N_{\mathrm{it}}^{s}}N_{\mathrm{it}},$$

where $N_{\mathrm{it}}$ denotes the observed population, $y_{\mathrm{it}}^{s}$ is the TB cases in the standard population. Thus, the overall crude rate of TB cases is given by:

$$r=\sum_{i}^{n}\sum_{t}^{T}\frac{y_{\mathrm{it}}^{s}}{N_{\mathrm{it}}^{3}}$$

and the overall number of expected TB cases is defined by:

$$E=\sum_{i}^{N}\sum_{t}^{T}r_{\mathrm{it}}^{s}N_{\mathrm{it}}=\sum_{i}^{N}\sum_{t}^{T}\frac{y_{\mathrm{it}}^{s}}{N_{\mathrm{it}}^{s}}N_{\mathrm{it}}.$$

Our first space-time model formulations is based on the framework developed by Bernardineli *et al.,* ^23^ where the linear predictor $\eta_{\mathrm{it}}$ is:

$$\eta_{\mathrm{it}}=\mu +\phi_{i}+u_{i}+\left(\rho +\delta_{i}\right)\times t,$$ (6)

where $\phi_{i}+u_{i}$ follows the BYM specifications ^24^ (See Section 0.0.2) with spatial structure $\left(\phi_{i}\right)$ and unstructured random effects $\left(u_{i}\right),$ $\rho v_{t}$ is the global linear time trend, $v_{i}\delta_{i}$ is the interactive term between space and time. ^16^^,^^23^ The term $v_{t}$ represents a vector of temporal weights and the intercept μ quantifies the average TB rate in all the 10 regions. Since the risk takes the form $\theta_{\mathrm{it}}=\exp\left(\eta_{\mathrm{it}}\right)$ then:

$$\theta_{\mathrm{it}}=\exp\left(\eta_{\mathrm{it}}\right)=\exp\left[\mu +\phi_{i}+u_{i}+\left(\rho +\delta_{i}\right)\times t\right].$$

It follows that the Poisson mean is

$$\mu_{\mathrm{it}}=E_{\mathrm{it}}\exp\left[\mu +\phi_{i}+u_{i}+\left(\rho +\delta_{i}\right)\times v_{t}\right]$$

and logarithm of the mean is given by:

$$\log\left(\mu_{\mathrm{it}}\right)=\log\left(E_{\mathrm{it}}\right)+\mu +\phi_{i}+u_{i}+\left(\rho +\delta_{i}\right)\times t.$$

These formulations suggest that each spatial unit has its own time trend with a spatial intercept $\left(\mu +\phi_{i}+u_{t}\right)$ and a slope $\left(\rho +\delta_{i}\right)$ . This model assumes a linear time trend in each spatial unit. The parameters to be estimated are $\varphi =\left\{\rho ,\phi ,u,\delta \right\}$ and the hyper-parameters $\psi =\left\{\tau_{\phi },\tau_{u},\tau_{\delta }\right\}$ .

Adjusting for risk factors $X_{i}$ of TB cases detection, the model 6 can be written as model 7. Now the parameters to be estimated are $\varphi =\left\{\beta ,\rho ,\phi ,u,\delta \right\}$ and the hyper-parameters are $\psi =\left\{\tau_{\phi },\tau_{u},\tau_{\delta }\right\}$ .

$$\eta_{\mathrm{it}}=\mu +\sum \beta_{i}X_{i}+\phi_{i}+u_{i}+\left(\rho +\delta_{i}\right)\times t$$ (7)

It is known that if $\delta_{i}<0$ the region-specific trend is less steep than the mean trend. On the other hand, $\delta_{i}>0$ implies that the region-specific trend is steeper than the mean trend. Further, $\delta_{i}∼\text{Normal}\left(0,\tau_{\delta }\right).$

The second space-time model is based on Waller *et al.* ^30^ dynamic non-parametric formulation on the linear predictor:

$$\eta_{\mathrm{it}}=\mu +\phi_{i}+u_{i}+\vartheta_{t}+\omega_{t},$$ (8)

where the terms $\mu ,\phi_{i},u_{i}$ follow the same formulation as in the first model. $\vartheta_{t}$ and $\omega_{t}$ structures denote the temporally structured and unstructured random effect, respectively. This model assumes a non-parametric time trend. Covariates are incorporated into Model 8 to estimate $\varphi =\left\{\mu ,\beta ,\phi ,u,\vartheta ,\omega \right\}$ and $\psi =\left\{\tau_{\phi },\tau_{u},\tau_{\vartheta },\tau_{\omega }\right\}.$ The model with the covariates can now be written as:

$$\eta_{\mathrm{it}}=\mu +\sum \beta_{i}X_{i}+\phi_{i}+u_{i}+\vartheta_{t}+\omega_{t}$$ (9)

The $\vartheta_{t}$ quantifies temporal-structure effect and it is modeled using a random walk through a neighboring structure ^16^ defined as:

$$\left\{\begin{array}{ll} \vartheta_{t}|\vartheta_{-t}∼N\left(\vartheta_{t+1},\tau_{\vartheta }\right) & t=1\\ \vartheta_{t}|\vartheta_{-t}∼N\left(\frac{\vartheta_{t-1}+\vartheta_{t+1}}{2},\frac{\tau_{\theta }}{2}\right) & t=2,\ldots ,T-1\\ \vartheta_{t}|\vartheta_{-t}∼N\left(\vartheta_{t-1},\tau_{\vartheta }\right) & t=T. \end{array}\right.$$

Finally $\phi_{t}$ is specified by means of a Gaussian exchangeable prior: $\omega_{t}∼N\left(0,\tau_{\omega }\right)$ . Finally $\phi_{t}$ is specified by means of a Gaussian exchangeable prior: $\omega_{t}∼N\left(0,\tau_{\omega }\right)$ .

The third space-time Model 10 is an extension of Model 9 that enables a space-time interaction in order to explain the difference in the time trend of TB cases. It is expressed as follows:

$$\eta_{\mathrm{it}}=\mu +\phi_{i}+u_{i}+\vartheta_{t}+\omega_{t}+\pi_{\mathrm{it}}$$ (10)

In this model, $\varphi =\left\{\mu ,\phi ,u,\vartheta ,\omega ,\pi \right\}$ and $\psi =\left\{\tau_{\phi },\tau_{u}\tau_{\vartheta },\tau_{\omega }\tau_{\pi }\right\}$ were estimated, where $\pi_{\mathrm{it}}$ is interaction between $\phi_{i}$ and $u_{i}.$ The model assumes that there is no interaction between $\phi_{i}$ and $\vartheta_{t},$ hence, $\pi_{\mathrm{it}}∼N\left(0,\tau_{\pi }\right).$ Incorporating covariates into Model 10, yields Model 11:

$$\eta_{\mathrm{it}}=\mu +\sum \beta_{i}X_{i}+\phi_{i}+u_{i}+\vartheta_{t}+\omega_{t}+\pi_{\mathrm{it}}.$$ (11)

Hence, $\theta =\left\{\mu ,\beta ,\phi ,u,\vartheta ,\omega ,\pi \right\}$ and $\psi =\left\{\tau_{\phi },\tau_{u}\tau_{\vartheta },\tau_{\omega },\tau_{\pi }\right\},\text{needtobeestimated}.$ For the interaction term $\pi_{\mathrm{it}}$ , it is assumed that there is spatial or temporal structure on the interaction, then $\delta_{\mathrm{it}}∼N\left(0,\tau_{\delta }\right)$ . ^37^ In this study, all the precision parameters are assumed to follow the gamma distribution. ^16^

## Results

In this section, the TB data has been analysed with the hierarchical space and space-time models.

Moreover, accuracy experiments for the space-time models using the Deviance Information Criterion (DIC) developed by Spiegelhalter was performed, in order to ascertain the most accurate model for predictive studies. In the discussion, only results obtained from significant predictors are reported and discussed. Further, in the analysis, a risk value higher than 1 is classified as high risk while risk lower than 1 is classified as low risk. Risk is classified as normal if it has a value of 1.

Furthermore, the space-time models discussed, involve the classical parametric framework ( 7) (presented by L. Bernardinelli *et al.*(1995) ^23^), the dynamic nonparametric framework presented by L. Knorr-Held *et al.* (2000) ^37^ for the linear predictor Equations (9), and Model ( 10). Model ( 10) (is an extension of Model ( 9)) to incorporate interactions between space and time. This enables us to explain the differences in the time trend of the TB cases across the regions. Equation (7) is refered to as **Model I**, Equation (9) as **Model II** and Equation (10) as **Model III**. Results are reported for experiments that involve adjustment and non-adjustment of covariates.

### Spatial BYM without covariates adjustment

**Figure 6.**
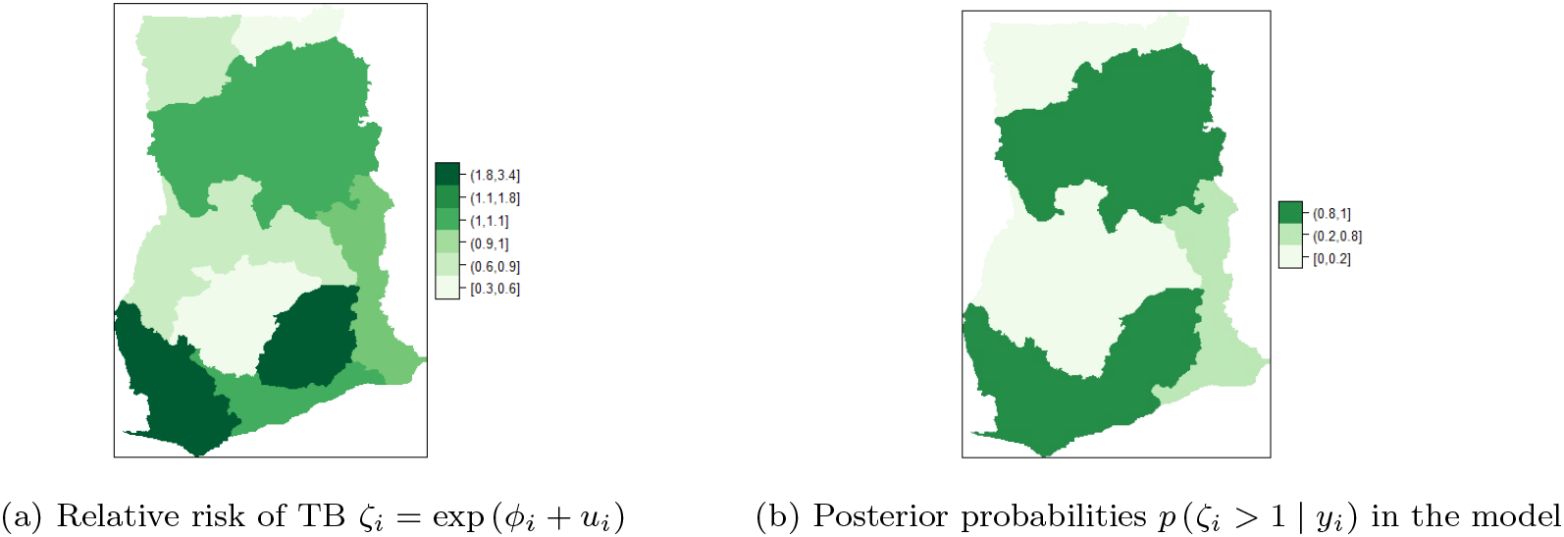
Regional distribution of posterior relative risk of TB in Ghana, using BYM.

In this section, BYM without covariate adjustments (defined as $\eta_{i}=\mu +\phi_{i}+u_{i}.$ ) was implemented. The posterior estimates are presented in Table 1. The maps of the posterior mean for the region-specific relative risks $\zeta_{i}=\exp\left(\phi_{i}+u_{i}\right)$ of presented in Figure 6a, b are used to identify regions with high risk. High risk is visualized by computing $p\left(\zeta_{i}>1|y\right),$ for details see M. Blangiardo *et al.* ^16^ Figure 6a shows the five of the ten regions that have high risk of TB cases. The risk profile for TB in Ghana are shown in the figure-legends. In the regional map of Ghana, the darker the region the higher the risk and vice versa. It can be observed that Upper East and Upper West Regions have the highest risk (with values in the range **1.8-3.4**), followed by Volta, Western and Central Regions with risk between **1.1-1.8**. The rest of the regions have low risk of TB detection, specifically, Northern and Ashanti Regions have the lowest risk (with values in the range **0.3-0.6**), followed by Greater Accra and Brong Ahafo Regions with values between **0.6-0.9**. The Eastern Region has normal risk (with a value in the range **1-1.1**).

**Table 1.** Summary statistics: posterior mean, standard deviation (Sd) and 95% credible interval for the fixed and random effects of the BYM.

	Estimate	sd	25%	50%	95% CI
**Fixed effects**					
μ	3.022	3.25	2.21	3.02	4.66
**Random effects**					
$\tau_{u}$	4.00	1.80	1.45	3.69	8.37
$\tau_{\phi }$	78.21	84.74	3.28	51.49	306.00

Figure 6b shows that Upper East, Upper West, Volta and Central regions have the highest posterior relative risk ( **0.8-1**) of TB detection. The regions have similar risks, however, none of them has a risk higher than the national risk 1. Moreover, low relative risk ( **0-0.2**) of TB detection is observed in the Northern, Brong Ahafo, Ashanti, and Greater Accra regions, followed by Eastern and Western regions that have relative risk values in the range ( **0.2-0.8**).

The results in Table 1 confirm the similarity or clustering of risk in the neighboring regions. This is indicated by the low variability captured by the precision of the spatial structure $\tau_{\phi }$ . The estimate of the posterior marginal variance to capture the amount of variability explained by the spatial structure was evaluated. The spatial structure effect empirically using this formula was estimated:

$$s_{\phi }^{2}=\frac{\sum_{i=1}^{n}{\left(\phi_{i}-\bar{\phi }\right)}^{2}}{n-1}$$ (12)

(where $\bar{v}$ is the average of ϕ ) and compare with the posterior marginal variance for the unstructured effect, provided by $\sigma_{u}^{2}:$

$${\text{frac}}_{\text{spatial}}=\frac{s_{\phi }^{2}}{\left(s_{\phi }^{2}+\sigma_{u}^{2}\right)}.$$

The estimated proportion of spatial structure variance is approximately 10%, implying that only 10% of the variability is explained by the spatial structure. It further explains the remaining higher variability captured by the unstructured random effect $u_{i}$ component of the BYM. The precision $\tau_{u}$ of the unstructured component of the BYM model indicates that the risk is heterogeneous among regions. The exponent of the posterior mean μ (overall mean effect) shows that there is approximately a 3-fold increase in TB infections rate across the 10 regions of Ghana. The corresponding 95% credible interval ranges from 2.21 to 4.66 .

### BYM with covariate adjustments

This section represents the results obtained from experiments conducted with seven (7) covariates adjustments of the BYM:

$$\eta_{\mathrm{it}}=\mu +\sum_{i=1}^{7}\beta_{i}X_{i}+\phi_{i}+u_{i}.$$

**Figure 7.**
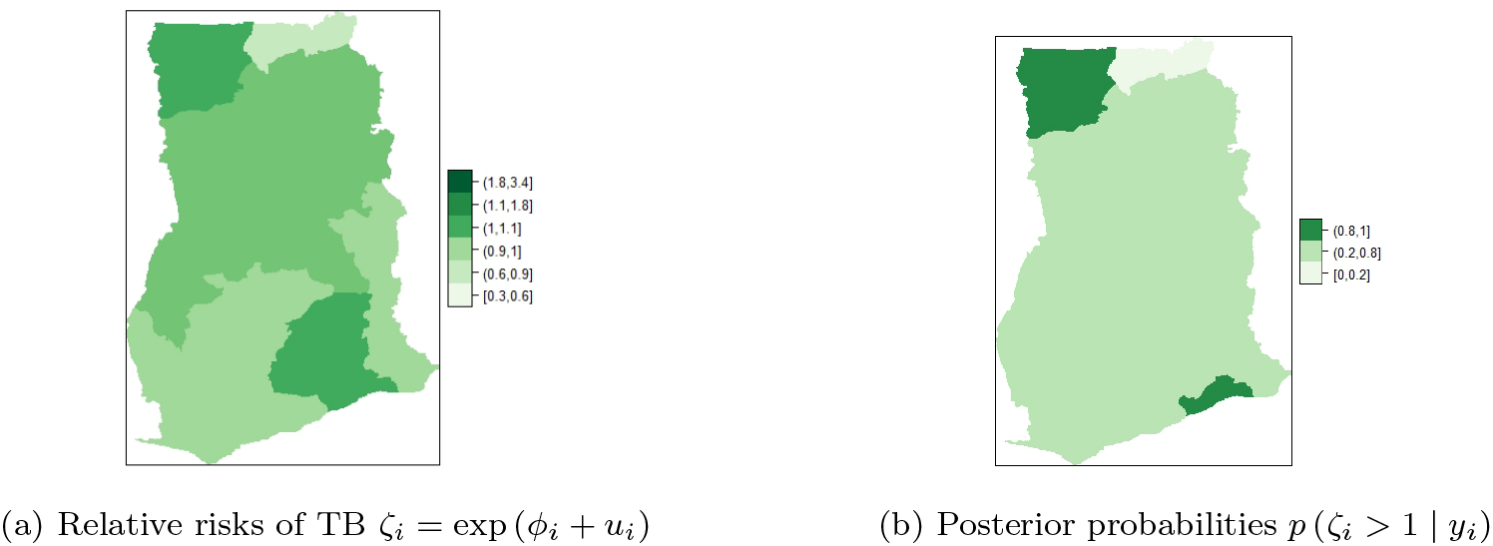
Regional posterior relative risk of TB in Ghana, using BYM with adjusted covariates.

Among the baseline predictors stated in Section b, the significant predictors for TB cases in Ghana that yield accurate models include: HIV prevalence, TB cure rate, TB success rate, proportion of people with knowledge about TB, proportion of those who know that TB is airborne, proportion in high income group and literacy *.* Table 2 presents posterior estimates of the overall mean, fixed effects (i.e. $\beta_{1},\ldots ,\beta_{7}$ ) as well as random effects (i.e. $\tau_{u}$ and $\tau_{\pi }$ ) for the unstructured and structured components of the BYM. The maps of the posterior mean for each region’s relative risk (i.e. $\zeta_{i}=\exp\left(\phi_{i}+u_{i}+\sum_{p=1}^{7}\mathrm{\beta p}\right)$ ) are presented in Figure 7a and b. The risk can be visualized by computing $P\left(\zeta_{i}>1|y\right)$ _._^16^

**Table 2.** Summary statistics: posterior mean, standard deviation (Sd) and 95% credible interval for the fixed and random effects of the BYM model.

	Estimate	Sd	25%	50%	95%
**Fixed effects**					
μ	9085.51	16.54	30.70	9115.48	2653723
$\beta_{1}$	1.081	1.027	1.024	1.081	1.141
$\beta_{2}$	0.855	1.040	0.789	0.855	0.927
$\beta_{3}$	1.046	1.017	1.010	1.046	1.083
$\beta_{4}$	0.897	1.055	0.806	0.897	1.000
$\beta_{5}$	0.450	1.297	0.266	0.450	0.762
$\beta_{6}$	0.946	1.017	0.914	0.946	0.979
$\beta_{7}$	1.116	1.045	1.022	1.116	1.219
**Random effects**					
$\tau_{u}$	25.80	20.62	3.83	20.41	80.01
$\tau_{\phi }$	1834.88	1810.43	121.58	1299.34	6656.65

It can be observed in Figure 7a that Upper East, Brong Ahafo and Western Regions have high and similar detection risks ranging from **1.1-1.8.** The adjusted risk (i.e. risk with the covariates in Figure 7a) is less than the unadjusted risk (risk without covariates in Figure 6a). Upper East Region is still among the regions with high risk of TB detection after covariates adjustment. Upper West Region does not belong to the high risk class while Brong Ahafo and Western Regions have moved to the normal risk class after the covariate adjustments. Greater Accra and Central Regions are the second highest in the high risk class, with risk ranging from **1-1.1**. Upper West, Northern, Volta, and Eastern Regions are in the normal risk class, with risk ranging from **0.9-1.** Further, Ashanti Region is in the low risk class (with values in the range **0.6-0.9**) after covariate adjustments. In Figure 7b, it can be observed that Brong Ahafo and Western Regions have the highest and similar relative risk ( **0.8-1**), while Ashanti Regions has the lowest relative risk ( **0.0-0.2**). The rest of the regions have similar relative risks ranging from **0.2-0.8**.

Table 2 presents the posterior estimates of fixed and random effects of the BYM with covariate adjustments. It can be observed that *TB cure rate* increases the risk of TB cases by approximately 8% This observation implies that as more cases are detected, more cases are cured and hence TB cases should in general decrease over time. This explains why TB success rate leads to 14% reduction in detection rates. The results also revealed that *knowledge about TB* significantly increases TB detection by approximately 5% This behaviour is expected because, as people become aware of TB, preventive measures are taken. *High income* is associated with 5% reduction in TB cases while *literacy* is associated with 12% increase in cases. *High income* increases the use of health facilities and testing for TB, thus, leading to a reduction of TB cases. *HIV prevalence* lead to 55% reduction in cases.

After adjusting the covariates, similarity/clustering of risk between neighboring regions, (see Figure 7) with low variability of risk among the regions was observed. This observation is captured by the precision $\tau_{\phi }$ of the spatial structure in Table 2. Heterogeneity of risk across the regions has reduced after the covariates adjustment.

Furthermore, the posterior marginal variance to determine the amount of variability explained by the spatial structure using the formulations in Equation (12) was evaluated. The results showed that the estimated proportion of spatial structure variance is approximately 5%. This implies that only 5% of the variability is explained by the spatial structure. Much of the variability remaining is captured by the unstructured random effect $u_{i}$ component of the BYM. The precision $\tau_{u}$ of the unstructured component of the BYM indicates that risk is heterogeneous across regions.

The posterior mean of the exponent μ (overall mean effect) gives an indication that there is approximately 9-fold increase in TB infections rate across the 10 regions in Ghana.

### Space-time models without covariates adjustments

Table 3 presents the DIC, mean deviance $\bar{D}$ and effective number of parameters pD components for the three space-time models. The performance indicators show that the classical parametric formulation (see Equation (7) introduced by Bernardinelli *et al*. ^23^ is the most accurate among the three space-time models. Hence, further discussion will include only the results from that model.

**Table 3.** Performance indicators for space-time models.

Model	$\bar{D}$	$p_{D}$	DIC
Model I	518.6	17.81	536.42
Model II	547.1	10.56	557.62
Model III	546.8	11.00	557.76

The results in Table 4 show that there is about 8% increase in risk of TB detection across the 10 regions of Ghana. However, this increase is statistically not significant at 5% significance level. As observed in the BYM, TB cases do not significantly increase with time. The precision parameter $\tau_{u}$ shows some level of variability in the risk of TB among the regions, while there is clustering of risk between neighboring regions exhibited by the high precision parameter $\tau_{\phi }$ for the spatial structure. High precision characterized by $\tau_{\delta }$ indicates low variability associated with $\delta_{i}.$ This further indicates that there is less interaction between space and time, as well as global trend ρ and areas-specific trend $\delta_{i}.$ Hence, the area-specific trend $\delta_{i}$ is less remarked than the mean trend.

**Table 4.** Summary statistics: posterior mean, standard deviation (Sd) and 95% credible interval for the fixed and random effects of the Model I.

	Estimate	sd	25%	50%	95% CI
**Fixed effects**					
μ	1.084	1.192	0.763	1.084	1.539
t	1.004	1.007	0.991	1.004	1.017
**Random effects**					
$\tau_{u}$	3.87	1.75	1.39	3.57	8.12
$\tau_{\phi }$	1847.48	1842.46	124.71	1301.61	6692.95
$\tau_{\delta }$	934.11	940.92	153.67	655.19	3397.82

**Figure 8.**
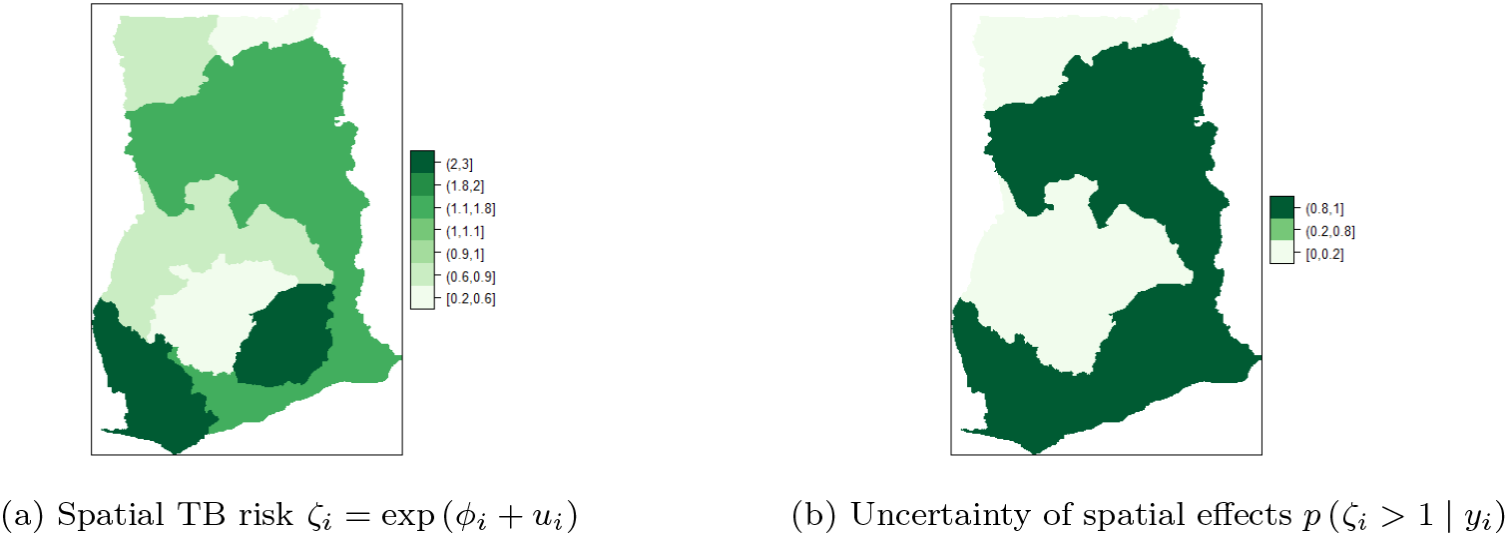
Spatial and uncertainty of TB risk, using space-time models.

Figure 8a shows the map of spatial trend $\zeta_{i},$ for the 10 regions and Figure 8b is the map of the posterior probabilities defined as $p\left(\zeta_{i}>1|y_{i}\right).$ Figure 8a shows that there is high risk of TB in the Eastern and Western regions. There is high risk (i.e. in the range **1.1-1.8**) and clustering among the neighboring regions; Volta, Northern, Central, and Greater Accra regions. Upper East and Ashanti regions have the lowest risk ( **0.2,0.6**) followed by Upper West and Brong Ahafo ( **0.6-0.9**). The results account for the high variability captured by the unstructured component and the low variability captured by the structured component of the area-specific trend. The posterior probabilities in Figure 8b indicate low risk (below 1) and relatively low level of associated uncertainty. ^16^ The time effect is not significant and there is no significant interaction between space and time. This observation accounts for the inaccuracy of **Model II** (i.e. Equation (10)) and **Model III** (i.e. Equation (9)) for the TB data.

**Figure 9.**
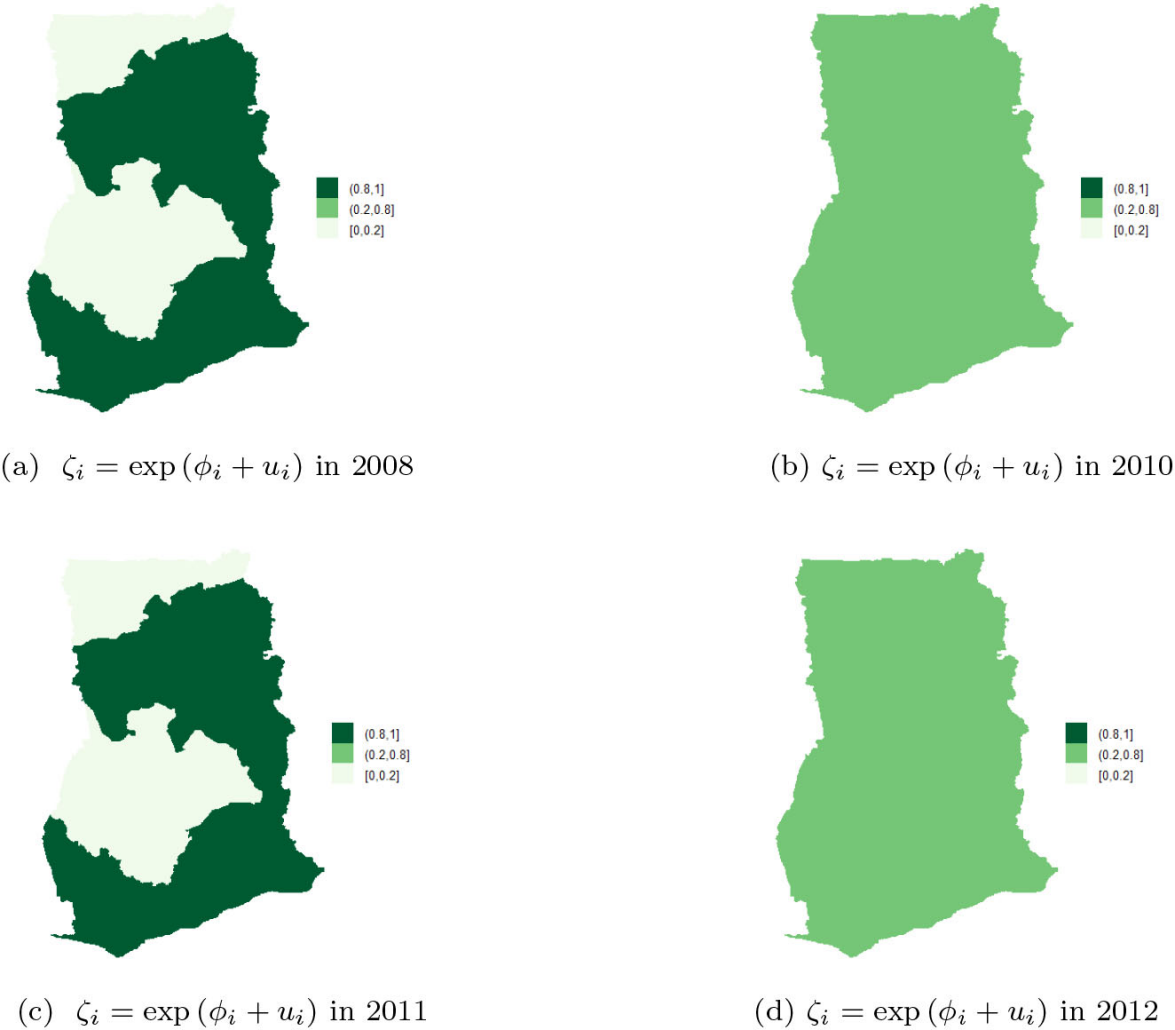
Spatial pattern of TB cases from 2008 to 2012.

Next, the posterior probabilities of each region by year was evaluated. The results indicate that time has no significant effect on the space-time pattern of TB cases as shown in Figure 9a- d. The risk of TB infection is almost the same across the 10 regions. Figure 9a shows that there is relatively high risks (in the range **0.8-1**) in the Northern, Volta, Eastern, Western, Central, and Greater Accra regions, while Upper East, Upper West, Brong Ahafo, and Ashanti regions have low relative risk (i.e. between **0-0.2**). The results exhibit clear clustering of risk among neighboring regions associated with low variability or uncertainty. In the year 2010, all the regions had risks in the range **0.2-0.8**, see Figure 9b. Similar observations can be made in year 2011 and the rest of the years shown in the figure. The results imply that it is sufficient to use only spatial models to estimate the risk of TB across the 10 regions of Ghana.

### Space-time models with covariate adjustments

It can be observed in Table 5, that **Model I** of the space-time models has the lowest DIC, mean deviance $\bar{D}$ and a high number of effective parameters pD . The indicators show that the classical parametric formulation of Ref. 23 (see Equation 7) is still the most accurate model among the three space-time models, for the TB data.

**Table 5.** Performance indicators of space-time models with adjusted covariates.

Model	$\bar{D}$	$p_{D}$	DIC
Model I	520.02	17.65	537.67
Model II	547.46	10.62	558.07
Model III	547.17	11.06	558.23

Table 6 shows negligible risk of TB across the 10 regions of Ghana. Similarly, the TB cases over time is statistically insignificant as observed previously. The precision parameter $\tau_{u}$ indicates very low variability in the risk of TB detection among the regions and much clustering of risk between neighboring regions exhibited by high precision parameter $\tau_{\phi }$ values for the spatial structure. High precision characterized by $\tau_{\delta }$ indicates lower variability associated with $\delta_{i}.$ This further indicates that there is no significant interaction between space and time as well as global trend ρ and areas-specific trend $\delta_{i}.$ Hence, the area-specific trend $\delta_{i}$ is less remarked than the mean trend.

**Table 6.** Summary statistics: posterior mean, standard deviation (Sd) and 95% credible interval for the fixed and random effects of the Model I.

	Estimate	sd	25%	50%	95% CI
**Fixed effects**					
μ	$3.65\times 10^{-6}$	3.50	$3.04\times 10^{-7}$	$3.67\times 10^{-6}$	$4.37\times 10^{-5}$
t	1.006	1.007	0.992	1.005	1.019
$\beta_{1}$	0.920	1.012	0.899	0.920	0.943
$\beta_{2}$	1.114	1.018	1.076	1.114	1.155
$\beta_{3}$	0.978	1.008	0.963	0.978	0.993
$\beta_{4}$	1.245	1.023	1.189	1.245	1.302
$\beta_{5}$	2.273	1.120	1.811	2.277	2.829
$\beta_{6}$	1.038	1.008	1.021	1.038	1.052
$\beta_{7}$	0.858	1.019	0.828	0.858	0.891
**Random effects**					
$\tau_{u}$	835.68	1220.69	2.33	337.68	4258.23
$\tau_{\phi }$	1272.83	1543.46	24.97	738.52	5456.29
$\tau_{\delta }$	521.70	400.33	114.09	412.56	1581.45

The results in Table 6 also revealed that *TB success rate* significantly increases TB cases by 11%. Also, *knowledge about TB* significantly reduces TB cases by approximately 2%, while increasing *TB cure rate*, significantly reduces detection by 8%. Awareness that *TB is airborne* increases TB detection by approximately 25%. That is, more people are willing to participate in TB testing to know their status leading to more case detections. It was also observed that *HIV prevalence* and *high income* significantly increases TB detection by 27% and approximately 4%, respectively. *Literacy* significantly reduces the risk of TB detection by approximately 14%.

**Figure 10.**
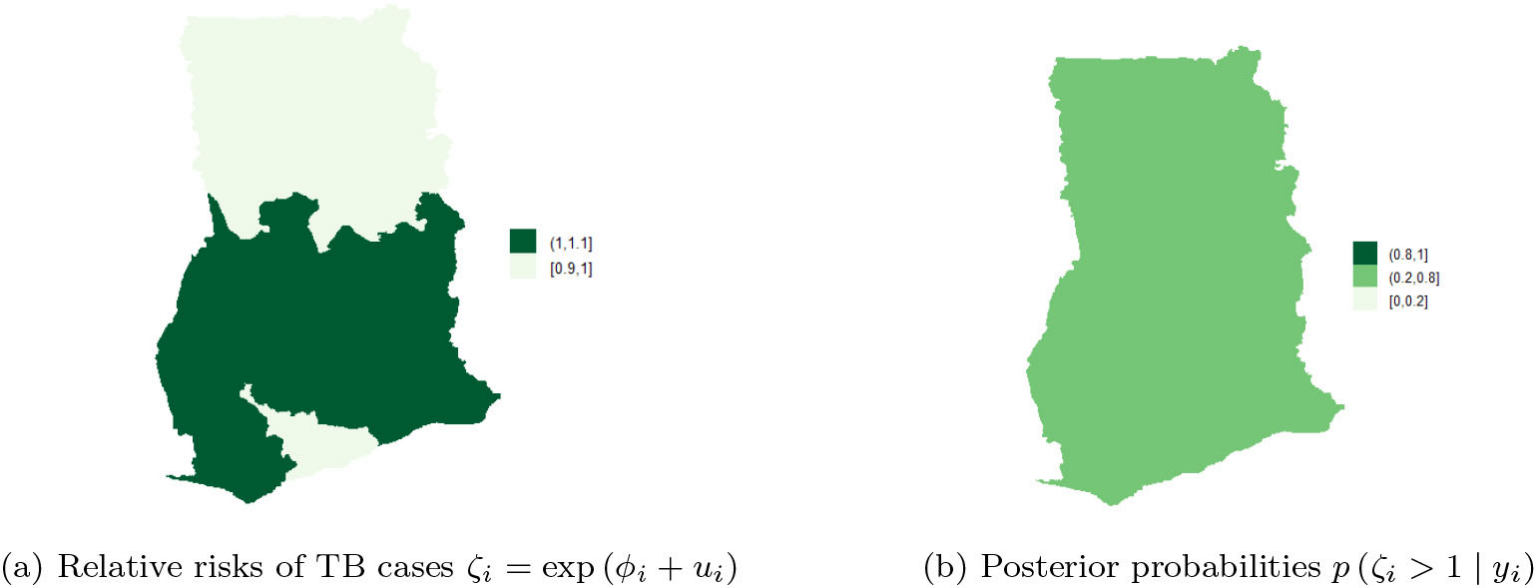
Posterior probabilities and relative risk of TB in the 10 regions of Ghana, using Model I with covariate adjustments.

Figure 10a shows the spatial trend $\zeta_{i}$ for the 10 regions and Figure 10b shows the posterior probabilities defined by $p\left(\zeta_{i}>1|y_{i}\right).$ Figure 10a shows that TB cases risk is higher and clustered in the Volta, Brong Ahafo, Ashanti, Eastern, Western and Greater Accra regions (ranging from **1-1.1**) while there is low risk in the Upper East, Upper West, Northern, and Central regions. Thus, there is high and similarity/clustering of risks among neighboring regions. After covariate adjustments, there is low risk $\left(0.6,1\right)$ in the Upper East, Upper West, Northern and Central regions. These observations account for the low variability captured by both the unstructured and structured components of the area-specific trend.

Figure 10b shows the posterior probabilities, it can be observed that all the regions have low relative risks (i.e. in the interval **0.2-0.8**) after adjusting the covariates. Since the time effect is negligible, there is no interaction between space and time. This observation accounts for the inaccuracy of **Model II** (10) and **Model III** (9) for the TB data. The posterior probabilities of the region after covariate adjustments (in the period 2008-2017), showed that the risks of TB across the 10 regions are the same/clustered in the range **0.2-0.8** (this observation is the same as the one made in Figure 10b).

## Discussion and conclusion

The spatial model used is based on the BYM ^24^ formulation. The results from this model (without covariate adjustments) showed that hot-spots of TB cases are located in five regions, i.e.; Upper East, Upper West, Volta, Western, and Central regions. Northern, Ashanti, Greater Accra, Brong Ahafo, Eastern and Western regions have low risk of TB detections. Another notable finding is the clustering of risk between neighbouring regions (i.e. nearby regions have similar risk). The results also revealed that the unstructured component of BYM (that explains variability of risk among the regions) is significant because the spatial structure only explains a small proportion of risk variability among regions. Additionally, after covariate adjustments, the number of high risk regions reduced from five to three (i.e. Upper East, Brong Ahafo and Western regions). The posterior probabilities in the BYM (with and without covariate adjustments) showed that there is clustering of risk between regions.

Further, this study also revealed that *TB cure rate, TB success rate, knowledge about TB, awareness that TB is airborne, HIV prevalence, percentage of literacy, high income* are important predictors of TB detection across the 10 regions of Ghana. Heterogeneity/variability of the risk reduced across the regions after covariate adjustments. The reduction in heterogeneity is due to low variance of the unstructured component and clustering due to low variability of the spatial or structured component of the BYM. Clustering of risk is evident from Figure 7, where almost all the regions have similar risk.

Furthermore, the study showed that the classical parametric formulation (i.e. Equation (7) called **Model I**) is the most accurate space-time model for the TB data. This model yields the lowest DIC, lowest mean deviation and highest effective number of parameters with or without covariate adjustments. Hence, it was selected for further experiments. Results from this model show that the risk of TB does not significantly increase over time. There is some level of heterogeneity in risk over time indicated by the precision of the unstructured component. There is relatively high level of clustering among neighboring regions as well. The results shows that there is less interaction of risk between space and time, as well as global trend and area-specific trend. Hence, the area-specific trend is less remarked than the mean trend.

Clustering of risk is evident per the relative risk profile in Figure 8. The space-time model classifies Eastern, Western, Volta, Northern, Central and Greater Accra regions as the hot-spots of the disease over time. Three of the regions (i.e. Volta, Western and Central regions) are classified as high risk regions, by the BYM without covariate adjustments and the Model I without covariate adjustments. The posterior probability in Figure 8b clearly shows clustering of risk and low level of associated uncertainty. The posterior probabilities over the study period are shown in Figure 9a- d. The figures show that the risk of TB does not change over time.

Moreover, after covariate adjustments, statistical inferences remained unchanged and the classical parametric formulation (in Equation 7) remains the most accurate model for the TB data. The posterior summary statistics in Table 6 showed negligible risk of TB across the 10 regions. Precisions of both the unstructured and structured components indicate clustering of risk among the regions. Therefore, all the regions exhibit similar risk. There is no significant space-time interaction due to low variability captured by $\tau_{\delta }.$ The results identify the risk factors under the BYM as significant predictors of TB detection.

Therefore, our study has characterized the spatio-temporal pattern of TB in Ghana, using hierarchical space-time models. The key findings include the identification of hot-spots, significant baseline predictors, heterogeneity/clustering of risk across regions and insignificant dependence of TB risk on time.

## Data availability

Source data The data used in this study can be found in the following links: https://open.africa/dataset/4176f749-cfa8-4e32-9418-86cef78f9db6/resource/0bcf9b54-3e35-4543-95cd-fd4de953edff/download/factsfigures_2018.pdf, https://www.who.int/teams/global-tuberculosis-programme/data https://www.stoptb.org/static_pages/GHA_Dashboard.html.

## Author contribution

Conceptualization, Software. Formal analysis: AKI Methodology and Investigation: AKI, FKB Data curation, Writing (Original draft preparation), and Writing (Review and editing): AKI and EAA Validation.

## References

[1] WHO: The top 10 causes of death. February 2022.

[2] W. H. Organization: ; Global tuberculosis report 2020: executive summary.

[3] Rose DN : The relationship between tb and hiv infections. *Occupational Medicine (Philadelphia, Pa.)* 1994;9(4):575–587. 7878489

[4] WHO: Tuberculosis. February 2022.

[5] Amo-Adjei J Awusabo-Asare K : Reflections on tuberculosis diagnosis and treatment outcomes in ghana. *Archives of Public Health.* 2013;71(1):1–8. 10.1186/2049-3258-71-22 23971675 PMC3765431

[6] Osei E Oppong S Adanfo D : Reflecting on tuberculosis case notification and treatment outcomes in the volta region of ghana: a retrospective pool analysis of a multicentre cohort from 2013 to 2017. *Global Health Research and Policy.* 2019;4(1):1–13. 10.1186/s41256-019-0128-9 PMC6916450 31890895

[7] Osei E Oppong S Der J : Trends of tuberculosis case detection, mortality and co-infection with hiv in ghana: A retrospective cohort study. *PLoS One.* 2020;15(6):e0234878. 10.1371/journal.pone.0234878 32579568 PMC7313972

[8] G. H. Service: The health sector in ghana facts and figures 2018. April 2021. Reference Source

[9] G. H. Service: National tuberculosis programme, monitoring and evaluation. April 2021. Reference Source

[10] Aryee G Kwarteng E Essuman R : Estimating the incidence of tuberculosis cases reported at a tertiary hospital in ghana: a time series model approach. *BMC Public Health.* 2018;18(1):1–8. 10.1186/s12889-018-6221-z PMC6258486 30477460

[11] Abdul IW Ankamah S Iddrisu A-K : Space-time analysis and mapping of prevalence rate of tuberculosis in ghana. *Scientific African.* 2020;7:e00307. 10.1016/j.sciaf.2020.e00307

[12] Aronis JM Ferraro JP Gesteland PH : A bayesian approach for detecting a disease that is not being modeled. *PLoS One.* 2020;15(2):e0229658. 10.1371/journal.pone.0229658 32109254 PMC7048291

[13] Fouarge E Monseur A Boulanger B : Hierarchical bayesian modelling of disease progression to inform clinical trial design in centronuclear myopathy. *Orphanet J. Rare Dis.* 2021;16(1):1–11. 33407688 10.1186/s13023-020-01663-7 PMC7789189

[14] Lawson A Lee D ; Bayesian disease mapping for public health. *Handbook of statistics.* Elsevier;2017; Vol.36: pp.443–481.

[15] Otiende V Achia T Mwambi H : Bayesian modeling of spatiotemporal patterns of tb-hiv co-infection risk in kenya. *BMC Infect. Dis.* 2019;19(1):1–13. 10.1186/s12879-019-4540-z 31660883 PMC6819548

[16] Blangiardo M Cameletti M Baio G : Spatial and spatio-temporal models with r-inla. *Spatial and spatio-temporal epidemiology.* 2013;4:33–49. 23481252 10.1016/j.sste.2012.12.001

[17] Lawson AB : *Bayesian disease mapping: hierarchical modeling in spatial epidemiology.* CRC press;2018.

[18] Iddrisu A-K Amoako YA : Spatial modeling and mapping of tuberculosis using bayesian hierarchical approaches. *Open J. Stat.* 2016;06(3):482–513. 10.4236/ojs.2016.63043

[19] Iddrisu A-K Alhassan A Amidu N : Investigating spatio-temporal pattern of relative risk of tuberculosis in kenya using bayesian hierarchical approaches. *Journal of Tuberculosis Research.* 2018;06(02):175–197. 10.4236/jtr.2018.62017

[20] Rue H Martino S Chopin N : Approximate bayesian inference for latent gaussian models by using integrated nested laplace approximations. *Journal of the Royal Statistical Society: Series B (Statistical Methodology).* 2009;71(2):319–392. 10.1111/j.1467-9868.2008.00700.x

[21] Wolpert RL Ickstadt K : Poisson/gamma random field models for spatial statistics. *Biometrika.* 1998;85(2):251–267. 10.1093/biomet/85.2.251

[22] Best N Richardson S Thomson A : A comparison of bayesian spatial models for disease mapping. *Stat. Methods Med. Res.* 2005;14(1):35–59. 10.1191/0962280205sm388oa 15690999

[23] Bernardinelli L Clayton D Pascutto C : Bayesian analysis of space—time variation in disease risk. *Stat. Med.* 1995;14(21-22):2433–2443. 10.1002/sim.4780142112 8711279

[24] Besag J York J Mollié A : Bayesian image restoration, with two applications in spatial statistics. *Ann. Inst. Stat. Math.* 1991;43(1):1–20. 10.1007/BF00116466

[25] Miller HJ : Tobler’s first law and spatial analysis. *Ann. Assoc. Am. Geogr.* 2004;94(2):284–289. 10.1111/j.1467-8306.2004.09402005.x

[26] Ghana:April 2021. Reference Source

[27] Lawson AB Browne WJ Rodeiro CLV : *Disease mapping with WinBUGS and MLwiN.* Wiley;2003; vol.11.

[28] Kyung M Ghosh SK : Bayesian inference for directional conditionally autoregressive models. *Bayesian Anal.* 2009;4(4):675–706. 10.1214/09-BA425

[29] Ntzoufras I : *Bayesian modeling using WinBUGS.* John Wiley and Sons;2011; vol.698.

[30] Waller LA Gotway CA : *Applied spatial statistics for public health data.* Wiley-Interscience;2004; vol.368.

[31] Mariella L Tarantino M : Spatial temporal conditional auto-regressive model: A new autoregressive matrix. *Australian Journal of Statistics.* 2010;39(3):223.

[32] Zumla A Petersen E Nyirenda T : Tackling the tuberculosis epidemic in sub-saharan africa–unique opportunities arising from the second european developing countries clinical trials partnership (edctp) programme 2015-2024. *Int. J. Infect. Dis.* 2015;32:46–49. 10.1016/j.ijid.2014.12.039 25809755

[33] Clayton D Kaldor J : Empirical bayes estimates of age-standardized relative risks for use in disease mapping. *Biometrics.* 1987;43:671–681. 10.2307/2532003 3663823

[34] Knorr-Held L Besag J : Modelling risk from a disease in time and space. *Stat. Med.* 1998;17(18):2045–2060. 10.1002/(SICI)1097-0258(19980930)17:18<2045::AID-SIM943>3.0.CO;2-P 9789913

[35] Roza DLd Caccia-Bava MdCG Martinez EZ : Spatio-temporal patterns of tuberculosis incidence in ribeirão preto, state of são paulo, southeast brazil, and their relationship with social vulnerability: a bayesian analysis. *Rev. Soc. Bras. Med. Trop.* 2012;45(5):607–615. 10.1590/S0037-86822012000500013 23152345

[36] Waller LA Carlin BP Xia H : Hierarchical spatio-temporal mapping of disease rates. *J. Am. Stat. Assoc.* 1997;92(438):607–617. 10.1080/01621459.1997.10474012

[37] Knorr-Held L Raßer G : Bayesian detection of clusters and discontinuities in disease maps. *Biometrics.* 2000;56(1):13–21. 10.1111/j.0006-341X.2000.00013.x 10783772

