# Spatio-temporal characteristics of Tuberculosis in Ghana

**DOI:** 10.12688/f1000research.109053.4

**Published:** 2024-06-21

**Authors:** Abdul-Karim Iddrisu, Emmanuel A. Amikiya, Francis Kwame Bukari

**Affiliations:** 1 Mathematics and Staistics, University of Energy and Natural Resources, Sunyani, Brong Ahafo, +233, Ghana; 2 Department of Management Science, Ghana Institute of Management and Public Administration, Accra, Greater Accra, +233, Ghana

**Keywords:** Bayesian spatial and space-time models, Tuberculosis relative risk, baseline predictors and TB hot-spots.

## Abstract

**Background:**

Tuberculosis (TB) remains a key contributor to global mortality; ranks second as the most fatal infectious disease and seventh among the top ten causes of death in Ghana. There is insufficient literature regarding the utilization of Bayesian hierarchical models for examining the spatial and spatio-temporal dynamics of tuberculosis risk in Ghana.

**Aim:**

This study addresses this gap by determining TB hotspots regions in Ghana using the Bayesian modeling framework.

**Methods:**

TB data were obtained from the Ghana Health Service and National Tuberculosis Programme for the 10 administrative regions of Ghana, from 2008 to 2017. Spatial and spatio-temporal TB relative risk for each region were estimated under the Bayesian modeling frameworks. Maps for TB risks were created to visualized regions with TB hotspots. Model fitting and parameter estimation were conducted using integrated nested Laplace approximation via R version 4.3.2.

**Results:**

Among the baseline predictors, TB cure rate, TB success rate, knowledge about TB, human immunodeficiency virus (HIV) prevalence, percentage of literacy, and high income were found to be most significant predictors of TB risk in Ghana. We noted an increased risk of TB infection in the Northern zone and the Eastern and Greater Accra regions in the Southern zone. Spatio-temporal distribution of TB infection risk was predominantly concentrated in the Southern zone. Clustering of TB risk was observed among neighboring regions.

**Conclusion:**

Factors influencing tuberculosis (TB) risk in Ghana are TB cure rate, TB success rate, knowledge about TB, HIV prevalence, literacy rate, and income level. The risk distribution was mainly concentrated in the Southern zone, with clusters of TB risk observed among neighboring regions. To achieve a significant reduction in TB cases, it is essential to allocate resources to TB hotspots regions and also implement measures to control significant predictors of TB infection risk.

## Background

Tuberculosis (TB) is an infectious disease caused by the bacillus Mycobacterium tuberculosis. ^1^ It is a condition that transcends age, gender, race, and the health status of individuals worldwide. ^1^ In 2019, it was estimated that around 10 million people had TB, resulting in an estimated 1.2 million deaths among those without HIV infection. ^1^ Among adults aged 15 and above, males comprised 56% of global infections, while females accounted for 32% in the same age category. Children represented 12% of total cases, with approximately 8.2% of reported cases involving individuals with HIV. ^1^ The distribution of TB infections in 2019 varied across regions, with Africa contributing to 25% of cases, South-East Asia and the Western Pacific accounting for 44% and 18%, respectively. In the same year, the Eastern Mediterranean, America, and Europe reported 8.2%, 2.9%, and 2.5% of the total cases, respectively. ^1^

Nevertheless, the World Health Organization (WHO) has advocated for the development of tuberculosis (TB) vaccines. ^1^ In addition to vaccines, there are available treatments for TB patients, and reports suggest that approximately 85% of patients can be successfully cured with a six-month drug regimen. ^1^ According to available data, treatment drugs have played a crucial role in preventing over 60 million deaths from 2000 to 2020. ^1^ Collectively, there was a 9% reduction in cases between 2015 and 2019, and a further 2.3% reduction between 2018 and 2019. ^1^ Europe has successfully achieved a 19% reduction in cases and a 31% reduction in deaths between 2015 and 2019. In the same timeframe, Africa has seen a 16% reduction in cases and a 19% reduction in deaths. More comprehensive statistics on TB can be found in references. ^1^^–^^6^

While global tuberculosis (TB) reports show a declining trend in cases and deaths, the WHO’s targets for reduction from 2015 to 2020 have not been met. ^1^ Except for Europe, all other continents have fallen short of achieving the prescribed reduction levels. ^1^ Consequently, TB remains among the top 10 causes of death, particularly in Africa and Ghana, where it outranks HIV/AIDS. ^1^

Ghana has been affected by the respiratory disease and currently has challenges in eradicating TB. In response, the country implemented policies such as Directly Observed Therapy (DOT) and National Tuberculosis Programmes (NTPs) in 1994 to detect and treat TB. ^1^^,^^7^^,^^8^ The initiation of the NTP resulted in 100% DOTs coverage by 2005, leading to an increased detection of TB cases for treatment each subsequent year. ^9^^,^^10^ For example, the number of TB cases detected rose from 7,425 in 1996 to 15,286 in 2009. ^9^^,^^10^ A more in-depth exploration of TB statistics in Ghana can be found in references. ^1^^,^^7^^–^^13^ The incidence of tuberculosis (TB) has demonstrated a consistent decline, with figures decreasing from 45,000 cases in 2010 to 44,000 in 2017. Annually, approximately 44,000 individuals develop TB in Ghana, with an estimated 6,600 being children. The number of TB patients undergoing treatment ranged between 14,607 and 15,389 from 2010 to 2017, highlighting a pressing demand for increased resources dedicated to TB patient care. In 2013, the national TB prevalence was estimated at 290 cases per 100,000 population, indicating a burden fourfold higher than the WHO’s estimates for the same year (71 cases per 100,000 population). TB-related mortality is estimated at 10,000 deaths annually. Tuberculosis is the seventh leading cause of mortality in Ghana, contributing to 4.9% of all deaths and older males, aged above 45 years, bear the greatest burden of the disease. Also, TB ranks 7th among the leading causes of death for females and 8th for males, and it stands as the 4th primary cause of mortality within the category of communicable, maternal, neonatal, and nutritional diseases. The TB epidemic exhibits a generalized pattern, albeit with geographical variations in case notification, often associated with disparities in access to healthcare facilities.

Despite a decline in TB cases and deaths attributed to the implementation of mitigation and treatment strategies, TB remains a life-threatening disease, placing a substantial burden on the health infrastructure in Ghana. Consequently, TB has become a focal point of research across diverse fields. Notably, researchers from various backgrounds, such as authors, ^7^^,^^8^^,^^14^ have investigated the dynamics of TB indicators and risk factors associated with TB in Ghana. Osei and colleagues, ^7^^,^^8^ studied the trends of TB detection and treatment outcomes, employing logistic regression to analyze the relationship between patient and disease characteristics. In a separate study, Aryee and colleagues ^14^ examined the dynamics of TB using Autoregressive Moving Average (ARIMA) methods, analyzing TB data recorded by Korle Bu Teaching Hospital from 2008 to 2017. On the other hand, Iddrisu and colleagues ^2^ investigated the temporal and geographical patterns of TB prevalence in Ghana between 2015 and 2018.

The existing body of literature on the utilization of the convolution model or the Besag, York, Mollié (BYM) model within the Bayesian modeling framework, particularly in the application of spatial and spatio-temporal models for estimating and mapping disease risk in Ghana, is limited. This paper addresses this gap by identifying regions with hotspots and predictors of TB infection risk in Ghana using these methods. We primarily focused on studying the spatial and spatio-temporal patterns of TB relative risk using spatial and spatio-temporal models within the Bayesian modeling framework. ^5^^,^^15^^–^^21^ These models enable the incorporation of neighboring regions’ information to yield accurate estimates of disease risk within and between regions. The key hypotheses guiding this study are as follows: (1) TB infection risk exhibits clustering among specific regions in Ghana, (2) there is variation in TB infection risk among different regions in Ghana, and (3) TB preventive measures, education on TB, and economic variables serve as significant predictors of TB infection risk in Ghana. Parameter estimates are derived from the marginal posterior distribution using the Integrated Nested Laplace Approach (INLA) implemented through R software version 4.3.2. ^20^^–^^23^

## Methods

This section provides a description of the data used in this study and specifies the Bayesian spatial and spatio-temporal models that were employed to estimate the relative risk of TB across the 10 old administrative regions of Ghana. The conditional auto-regressive (CAR) model and the random effect components of the Besag, York and Mollié (BYM) model for respectively identifying clustering and heterogeneity of disease risk in space and time are described. ^2^^,^^3^^,^^19^^,^^24^^–^^30^ The spatial model used is the BYM Model ^24^^,^^31^ and the spatio-temporal models are based on three modeling frameworks ^20^^,^^21^^,^^32^ developed by Knorr-Held and Ra’ser, ^33^ Bernardinelli and colleagues ^34^ and Waller and colleagues. ^35^ The best fitting spatio-temporal model was selected and only results from this model were discussed.

### TB cases

**Figure 1.**
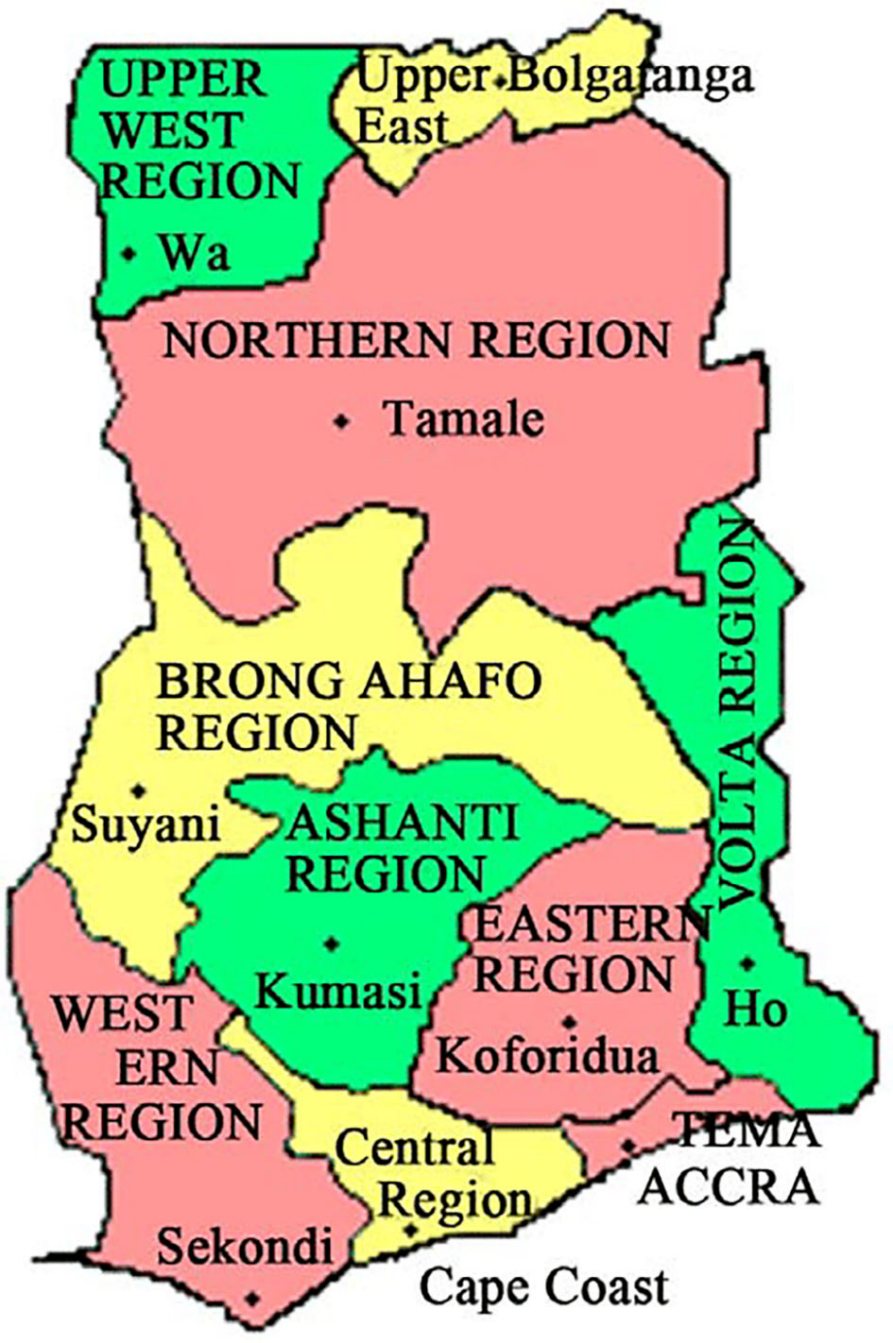
Map representing 10 administrative regions in Ghana.

For this study, we utilized tuberculosis (TB) detection data sourced from the Ghana Health Service and National Tuberculosis Programme. ^9^^,^^13^ The dataset comprises information on TB cases spanning the years 2008 to 2017, encompassing the 10 old administrative regions of Ghana. These regions consist of Ashanti, Brong Ahafo, Central, Eastern, Greater Accra, Northern, Upper East, Upper West, Volta, and Western, as illustrated in Figure 1. Although new regions were established after 2016, the TB data were collected and organized based on the original 10 administrative regions.

***Outcome variable***

The dependent variable in this study is the number of tuberculosis (TB) cases collected for each of the 10 administrative regions in Ghana, spanning the years 2008 to 2017.

### Baseline predictors

This study considered various baseline regional characteristics that may influence the risk of tuberculosis (TB) infection. The set of baseline predictors encompasses factors such as the doctor-to-population ratio, nurse-to-population ratio, HIV prevalence, tuberculosis cure rate, tuberculosis success rate, wealth quantiles, and the proportions of men and women who are employed, unemployed, educated, and uneducated. The study also considered variables such as the proportions of individuals who have heard about TB, possess knowledge that TB is airborne, are aware that TB can be cured, and believe that TB status should be kept confidential. In the data analyses, all these baseline variables are thoroughly examined to identify significant predictors of TB cases.

***Statistical methods***

In this section, we discussed and defined the spatial and spatio-temporal models employed for estimating tuberculosis (TB) risk in Ghana. Considering that these models are within the Bayesian modeling framework, we initially provided a concise overview of Bayesian principles.

***Bayesian modeling framework***

For spatial data collected at a single time point, where $y_{i},i=1,\ldots ,n$ represents the observed TB cases for region i , consider a Poisson random variable with a probability mass function defined as $\mathrm{Pr}\left(y_{i}|\theta \right)$ , where $\theta =\left(\theta_{1},\ldots ,\theta_{n}\right)$ represents a vector indicating the TB risk for each region. The likelihood function for the Poisson variable $y_{i}\;$ is defined as $\mathrm{Pr}\left(y|\theta \right)=\prod_{i=1}^{n}\;\mathrm{Pr}\left(y_{i}|\theta \right)$ , assuming that the sample values of the vector $y={\left(y_{1},\ldots ,y_{n}\right)}^{\prime }$ given the parameter estimates ***θ*** are independent. ^19^ In a Bayesian modeling framework, prior distributions of the unknown parameters ***θ*** in the likelihood function are required. The prior distribution reflects the existing knowledge about the parameters ***θ*** before observing the data $y_{i}$ . ^19^ Within the Bayesian framework, all parameters are stochastic and are assigned appropriate distributions known as prior distributions. ^19^

The Bayesian modeling framework integrates the likelihood function for the data with prior distributions for parameter estimates, giving rise to a probability distribution known as the posterior distribution. ^2^^,^^3^^,^^5^^,^^19^^–^^21^ This distribution, denoted as $\mathrm{Pr}\left(\theta |y\right)$ , signifies the probability distribution of the parameters given the observed data. It is proportional to the product of the likelihood function and the prior $\mathrm{Pr}\left(\theta \right)\;$ distributions, as defined by:

$$\mathrm{Pr}\left(\theta |y\right)=\frac{\mathrm{Pr}\left(y|\theta \right)\mathrm{Pr}\left(\theta \right)}{\int_{p}\;\;L\left(y|\theta \right)\mathrm{Pr}\left(\theta \right)d_{\theta }}$$ (1)

In equation (1), the denominated is often referred to as the normalizing constant and has been shown to integrate to 1. The implication is that the equation (1) can be alternatively expressed as $\mathrm{Pr}\left(\theta |y\right)\propto$ $\mathrm{Pr}\left(y|\theta \right)\mathrm{Pr}\left(\theta \right)$ . The Bayesian modeling framework assumes that the parameter estimates are stochastic and hence assumes a distribution for each parameter estimate, including hyperparameters. ^36^^–^^38^ The main criticism of such an assumption is that it depends on subjective prior assumptions, which might result in extreme or confusing conclusions. However, these priors also represent an advantage since they provide a tool for statisticians to be transparent about their assumptions. ^36^^–^^38^ Overweighting or informative priors have the potential to mislead statistical inferences about the data being studied since parameter estimates would be much influenced by such priors rather than the data under study. In Bayesian modeling, conjugate or non-informative priors are recommended in order to allow the data to have more influence on the parameter estimates. ^36^^–^^38^

Using traceplots or density plots of parameter estimates, ^39^ Markov Chain Monte Carlo (MCMC) methods determine when a model’s parameters have converged, greatly facilitating Bayesian inference for complex models. Nonetheless, these methods are computationally intensive and encounter various convergence issues. Integrated nested Laplace approximation (INLA) offers a computationally efficient alternative to MCMC for approximate Bayesian inference in latent Gaussian models. ^40^ These models encompass a broad range, from generalized linear mixed models to spatial and spatio-temporal models. ^20^^,^^40^ INLA employs a combination of analytical approximations and numerical algorithms for sparse matrices to approximate posterior distributions using closed-form expressions. This enables faster inference, avoid issues of sample convergence and mixing, thereby facilitating the analysis of large datasets and the exploration of alternative models. In this study, we obtained parameter estimates using INLA. ^40^ INLA, facilitated by the R-INLA package, has been widely embraced by various researchers. ^20^^,^^21^^,^^41^^,^^42^ Also, this package can directly be installed in R software by using the command install.packages(“INLA”,repos=c (getOption(“repos”),INLA=“ https://inla.r-inla-download.org/R/stable”), dep=TRUE).

***Spatial model: Besag, York and Mollié (BYM) Model***

This research utilizes the Besag, York, and Mollié (BYM) Model as its spatial modeling approach. ^3^^,^^19^^–^^21^^,^^24^^,^^31^^,^^43^ The model consists of two main components: the conditional autoregressive (CAR) model, which captures and conveys information about the clustering of disease risk among neighboring regions, and the random effect component, which identifies heterogeneity or variability in disease risk among regions. The BYM model is defined as $\;\eta_{i}=\mu_{i}+\phi_{i}+u_{i}$ , where $\mu_{i}\;$ represents the mean, $u_{i}$ embodies the random effect structure, and $\phi_{i}\;$ signifies the CAR structure. It is assumed that the random effect component, $u_{i}$ , follows a Gaussian distribution.

A compelling feature of the Conditional Autoregressive (CAR) model is its capability to create a framework that facilitates the sharing of strength among neighboring regions. As a result, adjacent regions are inclined to display similar risks, whereas those that are more distant are more likely to exhibit variability in terms of the risk associated with the studied disease. ^44^ In this investigation, we employed distances or boundary information between regions to define neighborhood properties within the CAR model. ^18^^,^^19^^,^^27^

Let $\varphi =\left\{i=1,2,\ldots ,n\right\}\;$ represent the regions, and $N_{i}=\left\{j\in \varphi :i\in j\right\}$ identifies regions that share boundaries with region i . Additionally, let $\phi_{i},$ where i∈φ , have a stochastic distribution. It is assumed that i∈Ω follows a Gaussian distribution, as outlined by:

$$\phi_{i}|\phi_{j\neq i}∼N\left(\sum_{i\neq j}\;\;W_{\mathrm{ij}}\phi_{j},\tau_{\phi }^{2}\right),$$ (2)

where $W_{\mathrm{ij}}$ represents the spatial dependence parameter determining the impact of each observation on the Conditional Autoregressive (CAR) structure $\phi_{i},$ where $\phi_{j}$ encompasses all observations except $\phi_{i}$ , and $\tau_{\phi }^{2}$ signifies the precision of $\phi_{i}$ . The spatial dependence parameter $W_{\mathrm{ii}}\;$ is non-zero if j∈S . To prevent autocorrelation, we designate $W_{\mathrm{ij}}$ = 0. This is to avoid conducting regression involving any observation against itself. ^24^^–^^26^^,^^35^^,^^44^ The model specification (2) indicates that $\phi_{i}$ solely relies on a set of neighboring $\phi_{j}$ when the region j is part of the neighborhood set $N_{i}$ of $\phi_{i}$ . For an in-depth explanation of the CAR structure, refer to. ^24^^–^^26^^,^^35^^,^^44^

Given that $y_{i}$ follows a Poisson distribution with parameter $E_{i}\exp\left(\eta_{i}\right)$ , and $\mu_{i}=E_{i}\exp\left(\eta_{i}\right)$ , the Besag, York, and Mollié (BYM) model with covariates can be described as $\eta_{i}=X^{\prime }\beta +\phi_{i}+u_{i}$ . Here, $E_{i}=rN_{i}$ represents the expected number of TB cases in region i , with $r=\frac{\sum_{t=1}^{n}\;y_{i}}{\sum_{i=1}^{n}\;N_{i}}$ , where $N_{i}$ denotes the at-risk population in region i . Furthermore, X denotes a design matrix of covariates, and β represents a vector of parameter estimates associated with these covariates.

Given $\theta_{i}=\exp\left(\eta_{i}\right)$ , it implies that $\log\left(\theta_{i}\right)=\eta_{i}$ , where $\eta_{i}$ represents the linear predictor. Consequently, the relative risk for each region i can be expressed as $\theta_{i}=\exp\left(X^{\prime }\beta +\phi_{i}+u_{i}\right)$ , and the log-log link function can be defined as $\log\left(\mu_{i}\right)=\log\left(E_{i}\right)+\exp\left(X^{\prime }\beta +\phi_{i}+u_{i}\right)$ .

To estimate parameters from the Bayesian posterior distribution, it is necessary to have the likelihood function of the data, along with prior distributions for the Conditional Autoregressive (CAR) structure, random effect, and the regression coefficients β . The prior distributions for the random effects and regression coefficients are assumed to conform to a Gaussian distribution. Consequently, the resulting posterior distribution can be specified as follows:

$$\mathrm{Pr}\left(\theta ,\beta ,u,\phi ,\tau_{\beta }^{2},\tau_{u}^{2},\tau_{\phi }^{2}|y,E\right)\propto \mathrm{Pr}\left(y,E|\theta ,\beta ,u,\phi ,\tau_{\beta }^{2},\tau_{u}^{2},\tau_{\phi }^{2}\right)\mathrm{Pr}\left(\beta \right)\mathrm{Pr}\left(u\right)\mathrm{Pr}\left(\phi \right).$$

The hyper-prior precision parameters $\tau_{u}^{2},\tau_{\phi }^{2}$ and $\tau_{\beta }^{2}$ follow the Gamma distributions: $\tau_{u}^{2}∼$ Gamma $\left(0.5,0.005\right),\tau_{\phi }^{2}∼\text{Gamma}\left(0.5,0.005\right)$ and $\tau_{\beta }^{2}∼\text{Gamma}\left(0.5,0.01\right)$ repectively. The vector of regression coefficients, β , is governed by a Gaussian distribution specified as $\beta ∼N\left(0,\tau_{\beta }^{2}\right)$ . The estimated parameters, $\tau_{u}^{2}$ and $\tau_{\phi }^{2}$ , represent the precision variance parameters for u and ϕ respectively. They are utilized to gauge the level of variability among regions and the clustering of risk among neighboring regions. ^19^ Additionally, we empirically estimated the spatial structure effect as $\tau_{\phi }^{2}=\frac{\sum_{i=1}^{n}\;{\left(\phi_{i}-\bar{\phi }\right)}^{2}}{n-1}$ , where $\bar{\phi }$ is the average of ϕ . This empirical estimation was then compared to the posterior marginal variance for the unstructured effect, provided by $\tau_{u}^{2}$ , provided proportion $\text{spatial}=\frac{\tau_{\phi }^{2}}{\left(\tau_{\phi }^{2}+\tau_{u}^{2}\right)}$ . We utilized $\mathrm{Pr}\left(\theta_{i}>1|y_{i}\right)$ to identify regions with a high probability of demonstrating a risk greater than 1.

***Spatio-temporal models***

The TB case data utilized in this study were gathered over a period, and consequently, relying solely on spatial models may not suffice to capture and elucidate the spatio-temporal pattern of the relative risk of TB. This limitation arises because spatial models are confined to identifying heterogeneity and clustering of disease risk at a specific time point. In this section, we introduce three spatio-temporal models ^20^ designed to investigate the spatio-temporal pattern of disease risk. These models vary in their space-time interaction structures. ^20^^,^^21^^,^^33^^,^^35^^,^^45^

Let $y_{\mathrm{ij}}$ denotes observed TB cases in region i , year j . The distribution of TB cases is defined as $y_{\mathrm{ij}}∼\text{Poisson}\left(E_{\mathrm{ij}}\exp\left(\eta_{\mathrm{ij}}\right)\right)$ , where the unknown relative risk $\theta_{\mathrm{ij}}$ for region i at year j can be expressed as $\theta_{\mathrm{ij}}=\exp\left(\eta_{\mathrm{ij}}\right)$ and $E_{\mathrm{ij}}$ is the expected number of TB cases in region i at year j . We defined the crude rate of TB cases for region i at year j as $r_{\mathrm{ij}}^{s}=\frac{y_{\mathrm{ij}}^{s}}{N_{i}^{s}}$ and the expected TB cases in region i at year j is defined as $E_{\mathrm{ij}}=r_{\mathrm{it}}^{s}N_{\mathrm{ij}}=\frac{y_{\mathrm{ij}}^{s}}{N_{\mathrm{ij}}^{s}}N_{\mathrm{ij}}$ , where $N_{\mathrm{ij}}$ denotes the observed population, $y_{\mathrm{ij}}^{s}$ is the TB cases in the standard population. This means that the overall crude rate of TB is given by $r=\sum_{i}^{n}\;\sum_{j}^{J}\;\frac{y_{\mathrm{ij}}^{s}}{N_{\mathrm{ij}}^{3}}$ and the overall number of expected TB cases is defined by $E=\sum_{i}^{N}\;\sum_{j}^{T}\;r_{\mathrm{ij}}^{s}N_{\mathrm{ij}}=\sum_{i}^{N}\;\sum_{j}^{J}\;\frac{y_{\mathrm{ij}}^{s}}{N_{\mathrm{ij}}^{s}}N_{\mathrm{ij}}$ . ^3^^,^^2^

The first spatio-temporal model ^34^ presented in this study defines the linear predictor $\eta_{\mathrm{it}}$ as

$$\eta_{\mathrm{ij}}=\mu +\phi_{i}+u_{i}+\left(\varrho +\delta_{i}\right)\times j,$$ (3)

where $\phi_{i}+u_{i}$ component follow the BYM ^24^ model’s specification, ϱ×j is the global linear time trend, $j\times \delta_{i}$ is the interaction term between space and time defining the difference between ϱ and the area-specific time trend. ^20^^,^^46^ The variable j represents a vector of temporal weights, and the intercept μ quantifies the average TB rate across all 10 regions. The differential trend $\delta_{i}$ signifies the interaction between time and space. The relative risk is expressed as $\theta_{\mathrm{ij}}=\exp\left(\eta_{\mathrm{ij}}\right)$ , and the logarithm of the risk is $\log\left(\theta_{\mathrm{ij}}\right)=\eta_{\mathrm{ij}}.$ This implies that $\theta_{\mathrm{ij}}=\exp\left(\eta_{\mathrm{ij}}\right)=\exp\left[\mu +\phi_{i}+u_{i}+\left(\varrho +\delta_{i}\right)\times j\right]$ . Consequently, the Poisson mean is $\mu_{\mathrm{ij}}=E_{\mathrm{ij}}\exp\left[\mu +\phi_{i}+u_{i}+\left(\varrho +\delta_{i}\right)\times j\right]$ , and the logarithm of the mean is defined as $\log\left(\mu_{\mathrm{ij}}\right)=\log\left(E_{\mathrm{ij}}\right)+\mu +\phi_{i}+u_{i}+\left(\varrho +\delta_{i}\right)\times j$ . These formulations suggest that each spatial unit possesses its own time trend with a spatial intercept $\left(\mu +\phi_{i}+u_{i}\right)$ and a slope $\left(\varrho +\delta_{i}\right)$ . This model assumes a linear time trend in each spatial unit. The parameters to be estimated are $\varphi =\left\{\varrho ,\phi ,u,\delta \right\}$ , and the hyper-parameters are denoted as $\psi =\left\{\tau_{\phi },\tau_{u},\tau_{\delta }\right\}$ .

When accounting for TB risk factors $X_{i}$ , model (3) can be expressed as model (4). The parameter estimates vector is now denoted as $\varphi =\left\{\beta ,\varrho ,\phi ,u,\delta \right\}$ , and the hyper-parameters are represented by $\psi =\left\{\tau_{\phi },\tau_{u},\tau_{\beta },\tau_{\delta }\right\}$

$$\eta_{\mathrm{ij}}=\alpha +\sum_{p=1}^{P}\;\beta_{p}X_{i}+\phi_{i}+u_{i}+\left(\varrho +\delta_{i}\right)\times j$$ (4)

It is established that if $\delta_{i}<0$ , the region-specific trend is less pronounced than the mean trend. Conversely, $\delta_{i}>0$ indicates that the region-specific trend is steeper than the mean trend. ^20^^,^^46^ The parameter $\;\delta_{i}$ is assumed as $\delta_{i}∼\text{Normal}\left(0,\tau_{\delta }\right)$ .

The second spatio-temporal model ^35^ defines the linear predictor as

$$\eta_{\mathrm{ij}}=\alpha +\phi_{i}+u_{i}+\vartheta_{j}+\omega_{j}$$ (5)

where the combination of terms $\phi_{i}+u_{i}$ represents the BYM model, and the structures $\vartheta_{j}$ and $\omega_{j}$ signify the temporally structured and random effects, respectively. This model assumes a non-parametric time trend. By incorporating covariates, the model (5) transforms into:

$$\eta_{\mathrm{ij}}=\alpha +\sum \beta_{i}X_{i}+\phi_{i}+u_{i}+\vartheta_{j}+\omega_{j}$$ (6)

The parameters to be estimated encompass $\varphi =\left\{\alpha ,\beta ,\phi ,u,\vartheta ,\omega \right\}$ , with corresponding hyper-parameters denoted as $\psi =\left\{\tau_{\phi },\tau_{u},\tau_{\vartheta },\tau_{\omega }\right\}$ . The temporally structured effect, $\vartheta_{j}$ , is captured and modeled using a random walk through a neighboring structure. ^20^ Specifically, for j=1 , $\vartheta_{j}|\vartheta_{-j}∼N\left(\vartheta_{t+1},\tau_{\vartheta }\right)$ ; for j=2,…,J−1 , $\vartheta_{j}|\vartheta_{-j}∼N\left(\frac{\vartheta_{j-1}+\vartheta_{j+1}}{2},\frac{\tau_{\theta }}{2}\right)$ ; and for j=J , $\vartheta_{j}|\vartheta_{-j}∼N\left(\vartheta_{j-1},\tau_{\vartheta }\right)$ . Finally, $\omega_{j}\;$ is specified using a Gaussian exchangeable prior: $\omega_{j}∼\text{Normal}\left(0,\tau_{\omega }\right)$ .

The third spatio-temporal model (7) proposed by ^33^^,^^47^ is an extension of model (6) to allow for a space and time interaction $\left(\pi_{\mathrm{ij}}\right)$ to explain the difference in time trend of TB cases for various regions.

The third spatio-temporal model (7), proposed by Knorr-Held, ^33^^,^^47^ extends model (6) by introducing a space and time interaction $\left(\pi_{\mathrm{ij}}\right)$ . This interaction term is included to account for variations in the time trend of TB cases across different regions.

$$\eta_{\mathrm{ij}}=\mu +\phi_{i}+u_{i}+\vartheta_{j}+\omega_{j}+\pi_{\mathrm{ij}}$$ (7)

In this model, we estimate $\varphi =\left\{\mu ,\phi ,u,\vartheta ,\omega ,\pi \right\}$ and $\psi =\left\{\tau_{\phi },\tau_{u}\tau_{\vartheta },\tau_{\omega },\tau_{\pi }\right\}$ , where $\pi_{\mathrm{ij}}$ represents the interaction between $\phi_{i}$ and $u_{i}$ . It assumes no interaction between $\phi_{i}$ and $\vartheta_{t}$ ; hence, $\pi_{\mathrm{ij}}∼N\left(0,\tau_{\pi }\right)$ . By incorporating covariates into the model (7), we obtain model (8) and subsequently need to estimate $\theta =\left\{\mu ,\beta ,\phi ,u,\vartheta ,\omega ,\pi \right\}$ and $\psi =\left\{\tau_{\phi },\tau_{u}\tau_{\vartheta },\tau_{\omega },\tau_{\pi }\right\}$

$$\eta_{\mathrm{ij}}=\mu +\sum \beta_{i}X_{i}+\phi_{i}+u_{i}+\vartheta_{j}+\omega_{j}+\pi_{\mathrm{ij}}$$ (8)

Concerning the interaction term $\pi_{\mathrm{ij}}$ , if we assume the presence of spatial or temporal structure, then $\pi_{\mathrm{ij}}∼N\left(0,\tau_{\delta }\right)$ . ^33^ In this study, all precision parameters are assumed to follow the gamma distribution. ^20^

## Results

In this section, we described the outcome variable and then applied the spatial and spatio-temporal models to the TB data. These models were implemented in R version 4.3.2 using the Integrated Nested Laplace Approximation (INLA) method. ^20^^,^^42^ A comparison of the performance of the spatio-temporal models was conducted, and the best-performing model was selected based on the Deviance Information Criterion (DIC) developed by Refs. 20, 42, 48. The model with the lowest DIC was considered the most effective. We then presented and discussed the results obtained from both the BYM model and the best-fitting spatio-temporal model.

### Description of the outcome variable

**Figure 2.**
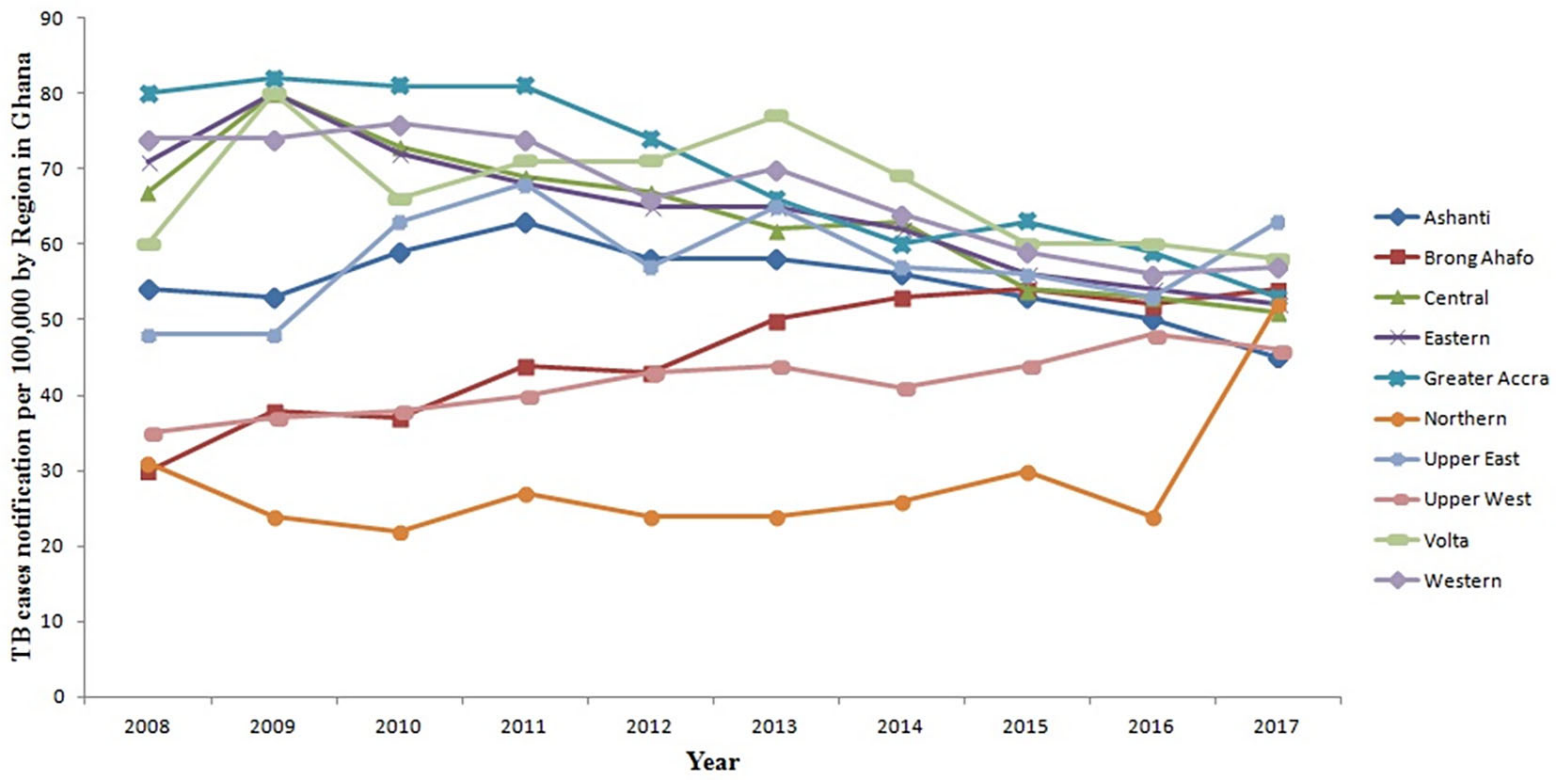
Trend of TB cases detection per 100,000 by region for 10 years from 2008 to 2017 in Ghana.

Figure 2 illustrates the TB trends in these regions over the specified time frame. Generally, there is an overall decline in TB cases observed across all regions of Ghana from 2008 to 2016, with the exception of the Brong Ahafo Region where TB cases increased. Notably, between 2016 and 2017, there was a significant increase in TB cases in the Northern and Upper East regions. Conversely, the Ashanti Region witnessed a decrease from 50 per 100,000 population in 2016 to 45 per 100,000 population in 2017. Specifically, TB cases in the Northern Region surged from 24 per 100,000 population in 2016 to 52 per 100,000 population in 2017. Similarly, the Upper East Region experienced an increase from 53 per 100,000 population in 2016 to 63 per 100,000 population in 2017. However, the changes in TB cases in the remaining regions remained relatively stable during this period.

**Figure 3.**
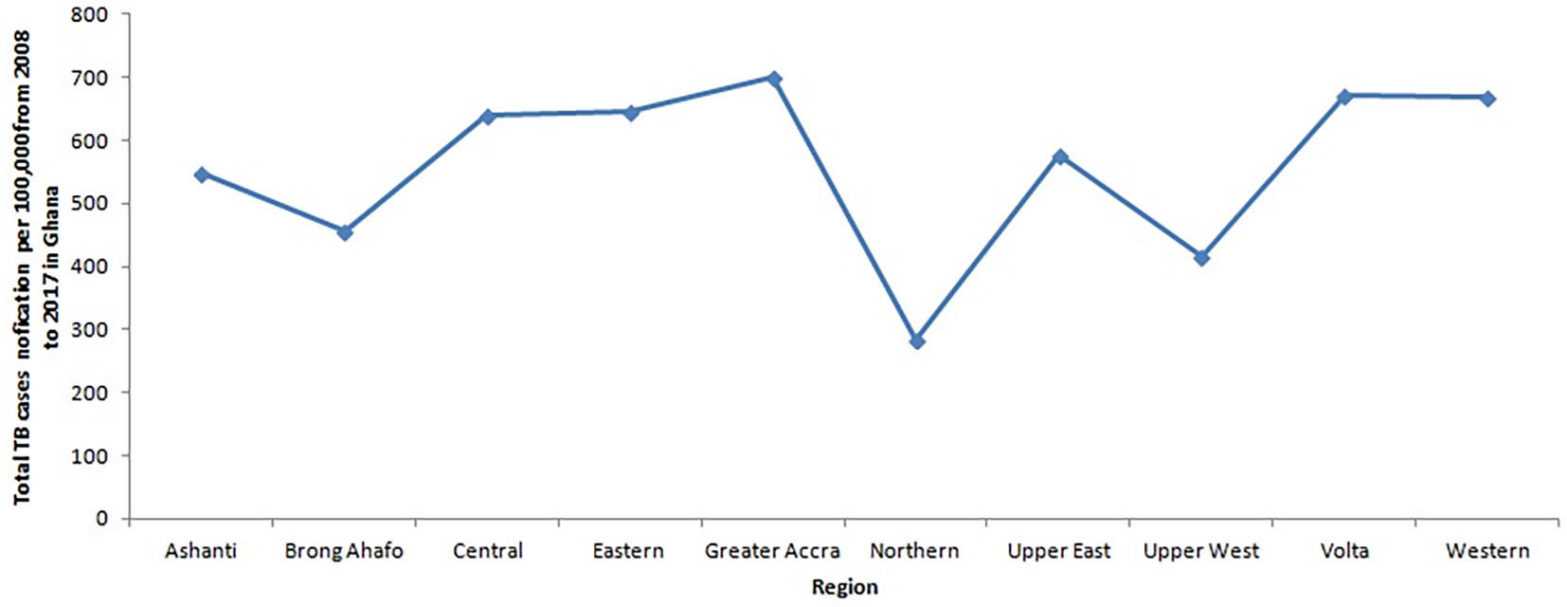
Trend of the total TB cases detection per 100,000 for each region from 2008 to 2017 in Ghana.

**Figure 4.**
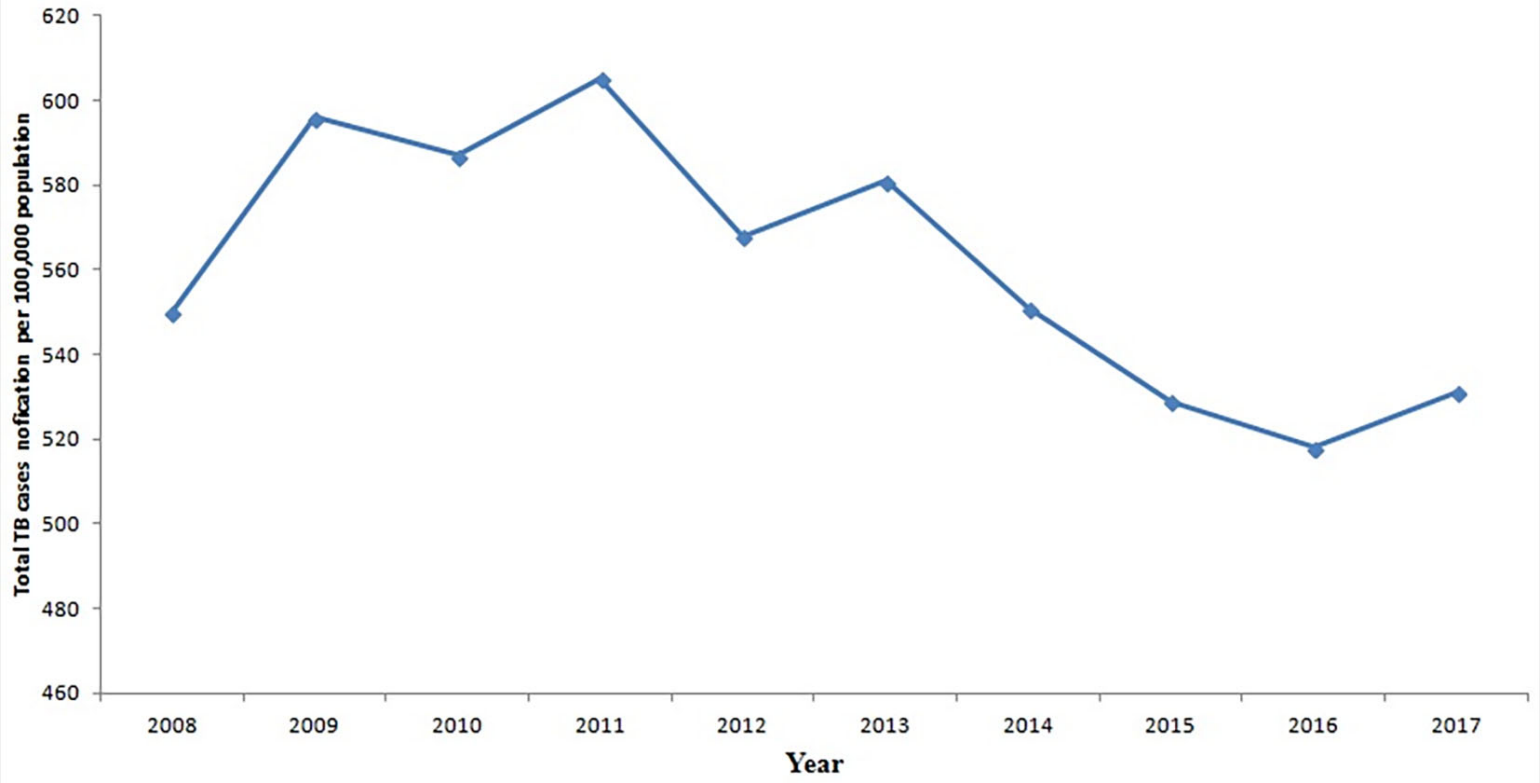
Trend of the total TB cases detection per 100,000 population for each year.

Figure 3 depicts the trajectory of the total number of tuberculosis (TB) cases for each region from 2008 to 2017. Notably, it illustrates that the Greater Accra Region consistently recorded the highest number of cases during this period, while the Northern Region registered the lowest. The Volta and Western Regions ranked second and third, respectively, with slightly lower case counts than the Greater Accra Region. Furthermore, Figure 4 displays the trend of total TB cases for each year or period. The data reveals that the peak in total TB cases occurred in 2011, whereas the lowest count was recorded in 2016. There is a discernible slow decline in TB cases from 2011 to 2017, marked by slight increases in 2012 and 2017.

**Figure 5.**
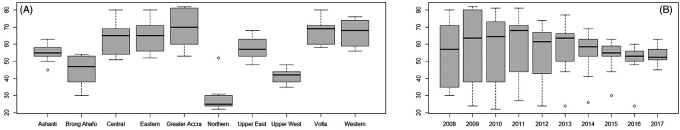
Box-and-whisker plot of TB cases by year (Figure A) and by region (Figure B).

Moreover, the variability of tuberculosis (TB) cases from 2008 to 2017 is illustrated through box-and-whisker plots in Figure 5. The overlapping box-and-whisker plots in Figure 5A suggest a lack of variability in TB cases across the years. These plots indicate a skew towards larger numbers from 2008 to 2014, shifting towards smaller numbers from 2015 to 2016. Particularly small numbers were noted in 2013, 2014, 2015, and 2017. Figure 5B presents the variability across regions. The non-overlapping box-and-whisker plots signify variability in TB cases among the regions. Additionally, it is observed that TB cases in most regions are skewed towards larger numbers, except for the Northern and Upper East regions.

***Results from the BYM model***

The results from the BYM model with the posterior estimates are shown in Table 1. Among the baseline predictors, the significant predictors for TB infection risk in Ghana that yield accurate models include: Tuberculosis cure rate $\left(\beta_{1}\right)$ , Tuberculosis success rate $\left(\beta_{2}\right)$ , proportion of people with knowledge about TB $\left(\beta_{3}\right)$ , proportion of those who know that TB is airborne $\left(\beta_{4}\right)$ , HIV prevalence $\left(\beta_{5}\right)$ , proportion in high income group $\left(\beta_{6}\right)$ and literacy $\left(\beta_{7}\right)$ .

**Table 1.** Summary statistics: posterior mean, standard deviation (Sd) and 95% credible interval for the fixed and random effects of the BYM model.

	Estimate	Sd	25%	50%	95%
**Fixed effects**					
*μ*	9.085	5.54	3.70	6.115	26.537
$\beta_{1}$	1.081	1.027	1.024	1.081	1.141
$\beta_{2}$	0.855	1.040	0.789	0.855	0.927
$\beta_{3}$	1.046	1.017	1.010	1.046	1.083
$\beta_{4}$	0.897	1.055	0.806	0.897	1.000
$\beta_{5}$	0.450	1.297	0.266	0.450	0.762
$\beta_{6}$	0.946	1.017	0.914	0.946	0.979
$\beta_{7}$	1.116	1.045	1.022	1.116	1.219
**Random effects**					
$\tau_{u}$	25.80	20.62	3.83	20.41	80.01
$\tau_{\phi }$	1834.88	1810.43	121.58	1299.34	6656.65

**Figure 6.**
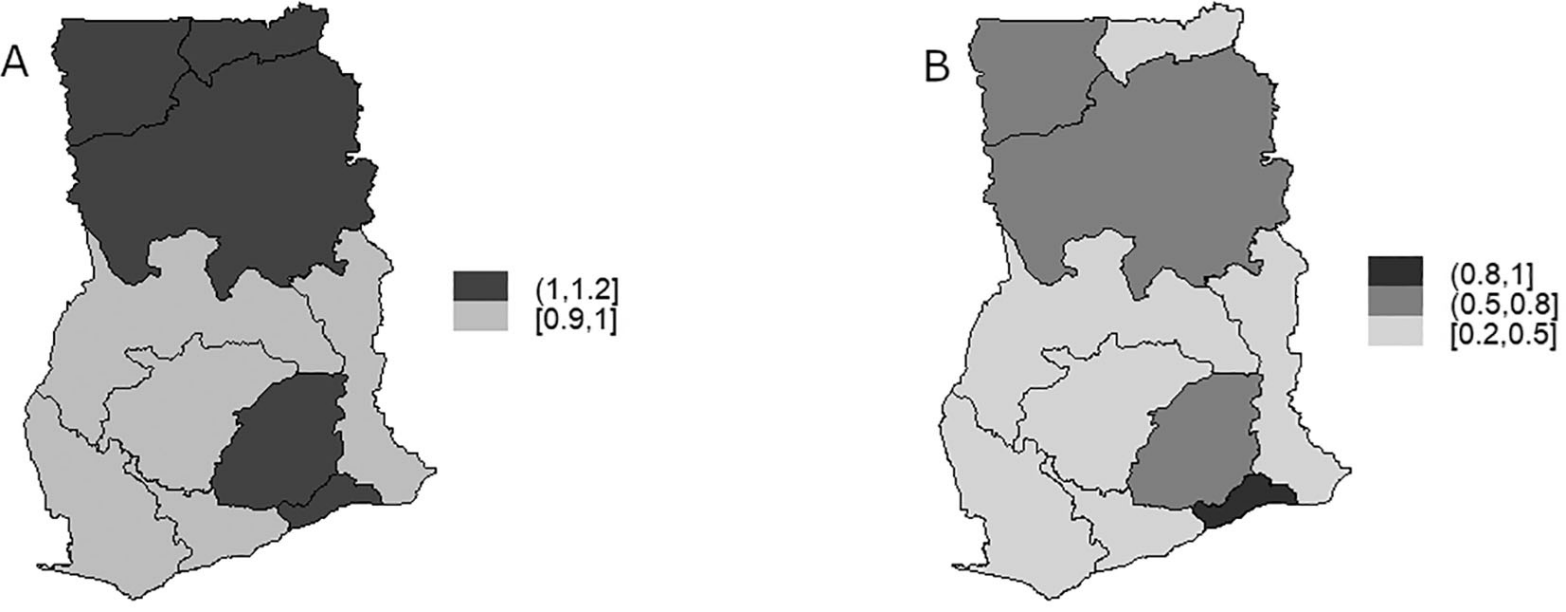
BYM model: Maps showing the distribution of the region-specific TB risk (Figure A) and distribution of region-specific exceedance probability (Figure B).

Figure 6A displays the map depicting the posterior mean for the region-specific relative risk $\left(\theta_{i}\right)$ of TB infection, while Figure 6B presents a map indicating the excess risk of TB. It can be observed Figure 6A that high risk of TB infection is distributed towards the Northern part of Ghana ranging between 1-1.2 for Upper East, Upper West and Northern regions. High risk of TB infection can be observed in Eastern and Greater Accra regions in the Southern part of Ghana. It can be observed in Figure 6B that Greater Accra region is the region with the highest probability of risk (0.8-1) exceeding the national risk 1 followed by Upper West, Northern and Eastern regions with risk between 0.5-0.8 and lowest risks (0.2-0.5) are observed in Upper East, Brong-Ahafo, Western, Volta, Ashanti, and Central regions.

Once more, there is slight variability in TB infection risk was noted across various regions, alongside significant clustering or similarities of TB infection risk among neighboring regions shown in Figure 6.

The results in Table 1 confirm the similarity or clustering of risk between or among neighboring regions. This is indicated by the low variability captured by the precision of the spatial structure $\tau_{\phi }$ . The precision $\tau_{u}$ of the unstructured component of the BYM model indicates that risk is slightly heterogeneous among regions. The exponent of the posterior mean μ (overall mean effect) indicates that there is approximately 9-fold reduction in TB infection rate across the 10 regions in Ghana.

From Table 1, It can be observed that TB cure rate $\left(\beta_{1}\right)$ increases the risk of TB infection by approximately 8% . This observation implies that as more cases are detected, more cases are cured and hence TB cases will in general decrease over time. This explains why TB success rate $\left(\beta_{2}\right)$ leads to 14% reduced risk of TB infection. The results also revealed that knowledge about TB $\left(\beta_{3}\right)$ significantly increases TB infection risk by approximately 5% . This behavior is expected because, as people become aware of TB, preventive measures are taken. The proportion of high-income group $\left(\beta_{6}\right)$ is associated with 5% reduction in TB infection while proportion of literacy $\left(\beta_{7}\right)$ is associated with 12% increase in TB cases. High income increases the use of health facilities and testing for TB, thus, leading to a reduction of TB cases. HIV prevalence $\left(\beta_{5}\right)$ in the region leads to a 55% reduction in TB cases.

***Best fitting spatio-temporal model for TB infection risk***

In this section, we fitted the three spatio-temporal models, adjusting for covariates effects on TB risk. Let us call the model (4); Model I, model (6); Model II and model (8); Model III. The best model is then selected and results from such a model are reported. Table 2 presents the DIC, mean deviance $\bar{D}$ and effective number of parameters pD components for the three space-time models. These indicators of a model’s performance suggest that Model I proved to be the best fitting model among the three candidates’ spatio-temporal models. Hence, we presented only the results from this model.

**Table 2.** Indicators of space-time models performance.

Model	$\bar{D}$	$p_{D}$	DIC
Model I	518.6	17.81	536.42
Model II	547.1	10.56	557.62
Model III	546.8	11.00	557.76

***Results from spatio-temporal model: Model I***

The results in Table 3 showed that TB infection risk decreases across the 10 regions of Ghana. However, there is no significant reduction in TB infection risk over the study period. The precision parameter $\tau_{u}$ indicates very low variability in the risk of TB detection among the regions and much clustering of risk between neighboring regions exhibited by high precision parameter $\tau_{\phi }$ values for the spatial structure. High precision characterized by $\tau_{\delta }$ indicates lower variability associated with $\delta_{i}$ . This further indicates that there is no significant interaction between space and time as well as global trend ϱ and area-specific trend $\delta_{i}$ . Hence, the area-specific trend $\delta_{i}$ is less remarked than the mean trend.

**Table 3.** Summary statistics: posterior mean, standard deviation (sd) and 95% credible interval for the fixed and random effects of Model I.

	Estimate	sd	25%	50%	95% CI
**Fixed effects**					
*μ*	0.177	1.075	0.020	0.181	1.400
t	1.006	1.007	0.992	1.005	1.019
$\beta_{1}$	0.920	1.012	0.899	0.920	0.943
$\beta_{2}$	1.114	1.018	1.076	1.114	1.155
$\beta_{3}$	0.978	1.008	0.963	0.978	0.993
$\beta_{4}$	1.245	1.023	1.189	1.245	1.302
$\beta_{5}$	2.273	1.120	1.811	2.277	2.829
$\beta_{6}$	1.038	1.008	1.021	1.038	1.052
$\beta_{7}$	0.858	1.019	0.828	0.858	0.891
**Random effects**					
$\tau_{u}$	835.68	1220.69	2.33	337.68	4258.23
$\tau_{\phi }$	1272.83	1543.46	24.97	738.52	5456.29
$\tau_{\delta }$	521.70	400.33	114.09	412.56	1581.45

The results also revealed that TB success rate significantly increases TB cases by 11% . Also, knowledge about TB significantly reduces TB cases by approximately 2% , while increasing TB cure rate, significantly reduces detection by 8% . Awareness that TB is airborne increases TB detection by approximately 25% . That is, more people are willing to participate in TB testing to know their status leading to more case detection. We also observed that HIV prevalence and high income significantly increases TB detection by 27% and approximately 4% , respectively. Literacy significantly reduces the risk of TB detection by approximately 14% .

**Figure 7.**
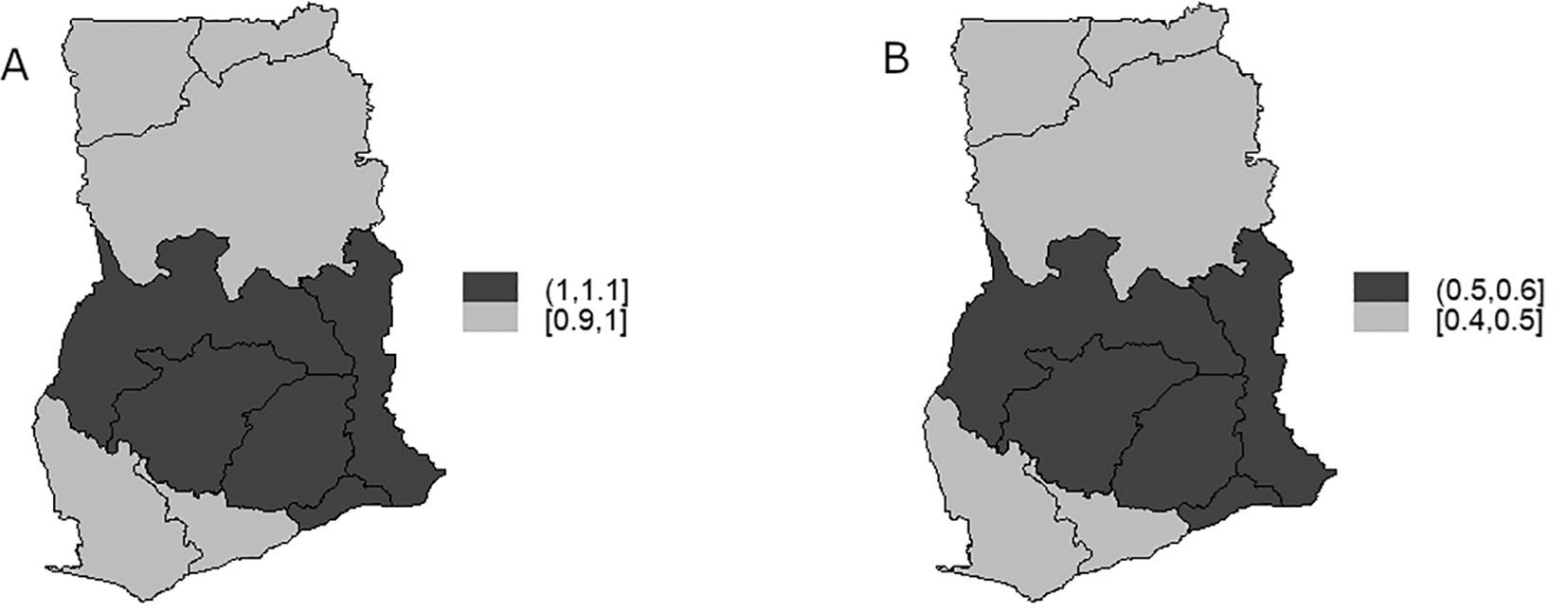
Spatio-temporal model: regions-specific risk (Figure A) and exceedance probability (Figure B).

**Figure 8.**
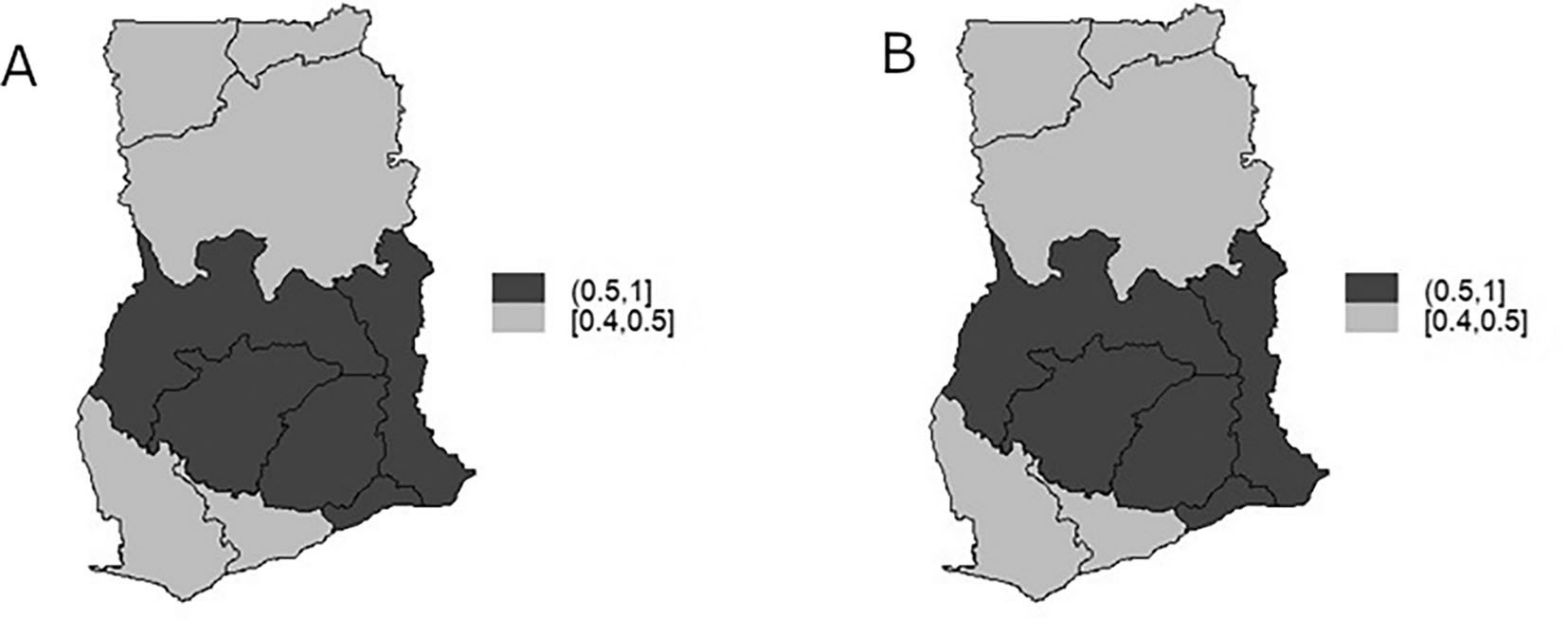
Spatio-temporal pattern of TB infection risk for 2008 (Figure A) and 2010 (Figure B).

The TB infection risk $\theta_{i}$ representing the spatial component of the spatio-temporal model is display in Figure 7A and Figure 7B is the map of exceedance risk. Both Figure 7A and Figure 7B showed that TB infection risk is clustered among regions in the Southern part of Ghana with Brong-Ahafo, Volta, Ashanti, Eastern, Greater Accra regions showing high risk (1-1.1) and the rest of the region show low risk between 0.9-1. These observations account for the low variability captured by both the unstructured and structured components of the area-specific trend. Furthermore, the precision $\left(\tau_{\vartheta }\right)$ of the random effect of the temporal structure $\left(\vartheta_{j}\right)$ suggests a clustering of the relative risk of TB over time. Maps showing spatio-temporal pattern of TB infection risk for 2008 and 2010 are respectively presented in Figure 8A and Figure 8B for illustration. Elevated TB infection was found in the Southern part of the country across all the years. However, the infection risk was consistently below 1 in all the years.

## Discussion

Accurate assessment and mapping of a disease risks are crucial for the efficient allocation of resources and targeted interventions to address disease hotspots, where there is a high risk of the disease. This research provides valuable insights into tuberculosis (TB) infection hotspots across various regions in Ghana by employing maps that highlight areas with elevated TB infection risk. The study investigates the spatial and spatio-temporal patterns of TB prevalence using a Bayesian modeling framework. Within this framework, we explore the clustering and heterogeneity of TB relative risk, utilizing a conditional auto-regressive (CAR) spatial structure and an unstructured component for random effects ^24^ respectively. The data utilized in our analysis are sourced from the Ghana Health Service and the National Tuberculosis Program. To fit the models, we employed the Integrated Nested Laplace Approximation through the R software. ^20^^,^^21^^,^^40^

In this study, the model utilized to explore the spatial pattern of TB risk was based on the convolution model, specifically the Besag-York-Mollié (BYM) model. ^24^ The findings indicate a heightened risk of tuberculosis (TB) infection in the Northern part of Ghana, specifically in the Upper East, Upper West, and Northern regions. Similarly, the Southern part, particularly the Eastern and Greater Accra regions, exhibits a high risk of TB infection. Notably, the Greater Accra region stands out with the highest probability of risk, surpassing the national risk level of 1, a trend consistent with the observation that more TB cases are reported in the Greater Accra regions, as noted by various authors. ^49^ Additionally, the study reveals significant clustering or similarities in the risk of tuberculosis (TB) infection among adjacent regions in Ghana. Moreover, there is an observed decline in the rate of TB infections across the ten regions in Ghana, as observed in Refs. 13, 50. The clustering of disease risk between regions may stem from shared geographic factors or possibly be linked to characteristics such as demographics, clinical aspects, or epidemiological factors. ^51^

The study findings indicate that the TB cure rate is associated with an increase risk of TB detection. This suggests that as more cases are detected and cured, the overall number of TB cases tends to decrease over time. Conversely, a higher TB success rate is linked to a reduction in the risk of TB infection, highlighting the effectiveness of successful treatment in lowering the prevalence of TB. Furthermore, increased knowledge about TB is shown to significantly raise TB infection risk; as more people are willing and available to be tested for TB, more TB infected cases are detected. On the socioeconomic front, a higher proportion of high-income individuals is associated with a reduction in TB infection, as it correlates with increased use of health facilities and testing for TB. However, a higher proportion of literacy is linked to an increase in TB cases, suggesting a complex relationship between literacy levels and TB infection. Notably, HIV prevalence is found to be associated with a substantial reduction in TB cases, underscoring the impact of HIV prevalence on TB infection rates. The reduced risk of TB infection may be due to the fact that regions with high prevelance of HIV might have received attention in terms of HIV counseling, screening for TB and treatment. For instance, interventions such as early HIV counselling and screening for TB patients and early diagnosis and initiation of antiretroviral therapy (ART) to coinfected individuals have all been shown to be effective in preventing TB disease. ^52^^,^^53^

We also studied the spatio-temporal distribution of TB infection risk using the spatio-temporal modeling frameworks proposed by Bernardinelli and colleagues, ^46^ Waller and colleagues ^35^ and Knorr-Held and Raßer. ^54^ To determine the most suitable spatio-temporal model, we fitted these models and employed their individual Deviance Information Criterion (DIC) for selection. Among these models, the space-time model with the interaction term proposed by Bernardinelli and colleagues ^46^ demonstrated the optimal fit to the TB data, as evidenced by its lowest AIC value. The findings indicated a decreased risk of TB infection across the country. Nonetheless, there was no significant reduction in TB infection risk over the study period, and there remained substantial clustering of risk among neighboring regions. High TB infection risk was identified consistently in the Southern part of the country throughout the years, although the infection risk consistently stayed below 1. Within the Southern part of Ghana, TB infection risk showed clustering among regions, with Brong-Ahafo, Volta, Ashanti, Eastern, and Greater Accra regions exhibiting elevated risk levels.

The data utilized in this study possesses a spatial dimension, encompassing reported TB cases across various districts, towns, and villages within the region. As a result, there are often shared factors ^55^ that contribute to the prevalence of TB. This highlights the importance of identifying regions with high TB incidence, and facilitating the allocation of essential resources to address variables recognized as potential contributors to TB infection in those specific areas. The significance of regional-level modeling in the context of TB decision-making in Ghana is considerable. By employing regional-level models, it becomes possible to comprehend the localized dynamics and specific factors contributing to TB prevalence within distinct regions of the country. This approach facilitates more targeted and effective decision-making in resource allocation, intervention strategies, and the deployment of healthcare measures. It provides a deeper understanding of the unique challenges and variations in TB transmission, ensuring that interventions are tailored to address the specific needs of different regions in Ghana.

There is a notable absence of comprehensive literature addressing the modeling and mapping of tuberculosis (TB) risk in Ghana, particularly in terms of statistical methodology and practical application. The current body of literature has predominantly focused on spatial and seasonal modeling and mapping of TB risk, ^49^^,^^56^ with no exploration of spatio-temporal risk across different years by various authors. While some studies have conducted modeling and mapping of TB risk without considering seasonal or spatio-temporal effects, ^14^ others have incorporated the seasonal pattern of TB risk. ^49^^,^^56^ Notably, there is a lack of studies investigating the yearly or monthly spatio-temporal effects on TB infection risk in Ghana. In general, the literature lacks an in-depth exploration of statistical methodologies and applications for modeling and mapping TB infection risk in Ghana, especially in the domain of spatio-temporal modeling using advanced statistical methods. This study aims to address this gap and contribute to the existing literature on the modeling and mapping of TB risk in Ghana. It serves as a pioneering effort in utilizing these statistical methodologies to examine spatial and spatio-temporal aspects of TB risk in the country. Emphasizing the use of such statistical methodologies in studying disease risk in Ghana, the study underscores their ability to provide precise estimates of disease risk in both space and time.

### Limitations

One significant limitation is the reliance on existing data, which could contain errors if healthcare workers input information incorrectly. Additionally, using secondary data may compromise external validity if biases exist in the population under study. Another potential source of bias is non-random participant selection, which could make the estimates unreliable if the sample doesn’t accurately represent the entire population.

## Conclusion

The identification of TB hot-spots in the Northern part of Ghana and the Greater Accra and Eastern regions in the Southern part of Ghana suggests a need for targeted interventions in these areas. Factors such as TB cure rate, TB success rate, knowledge about TB, income status, and HIV prevalence were identified as significant predictors of TB infection risk. This suggests that TB control measures should focus on treating more TB infected patients, creating TB awareness among the general public, making healthcare facilities available and accessible for both the poor and rich, and ensuring people living with HIV are identified early for treatment.

Similar levels of TB infection risk were observed among neighboring regions in Ghana, suggesting that these areas share common geographical traits influencing TB risk. This highlights the necessity for further research to identify such factors, enabling targeted interventions to enhance TB control measures.

Our study underscores the critical importance of precise risk assessment and mapping for effective resource allocation and targeted interventions. This research contributes valuable insights into disease hotspots across various regions in Ghana, utilizing advanced mapping techniques. The convolution model, incorporating both structured and unstructured components, facilitated spatial modeling with Bayesian methodology, revealing substantial variability and clustering of risk among neighboring regions. The findings unveiled distinct patterns of risk distribution, with certain regions exhibiting heightened risk over specific years.

In conclusion, our study enhances the understanding of TB dynamics in Ghana by employing sophisticated modeling techniques and innovative spatial and spatio-temporal analyses. These insights provide a foundation for informed decision-making, resource allocation, and targeted interventions, contributing to the ongoing efforts against TB and improving public health outcomes. Regions sharing similar risk patterns, possibly due to common local climatic conditions, can guide targeted resource allocation, given the regional basis of resource allocation in Ghana. The utilization of advanced statistical methods, encompassing both spatial and spatio-temporal analyses, is encouraged to further enrich the existing statistical literature on modeling and mapping TB risk in Ghana. We also recommend application of these methodologies to study spatial and spatio-temporal patterns of TB infection risk using TB data on all the 16 administrative regions in Ghana.

## Data availability

Source data The data used in this study can be found in the following links: https://open.africa/dataset/4176f749-cfa8-4e32-9418-86cef78f9db6/resource/0bcf9b54-3e35-4543-95cd-fd4de953edff/download/factsfigures_2018.pdf, https://www.who.int/teams/global-tuberculosis-programme/data https://www.stoptb.org/static_pages/GHA_Dashboard.html.

## Author contribution

Conceptualization, Software. Formal analysis: AKI Methodology and Investigation: AKI, FKB Data curation, Writing (Original draft preparation), and Writing (Review and editing): AKI and EAA Validation.

## References

[1] World Health Organisation : *Global tuberculosis report 2020: executive summary.* World Health Organization:2020.

[2] Iddrisu AK Alhassan A Amidu N : Investigating Spatio-Temporal Pattern of Relative Risk of Tuberculosis in Kenya Using Bayesian Hierarchical Approaches. *J Tuberc Res.* 2018;06(02):175–197. 10.4236/jtr.2018.62017

[3] Iddrisu AK Amoako YA : Spatial Modeling and Mapping of Tuberculosis Using Bayesian Hierarchical Approaches. *Open J Stat.* 2016;06(03):482–513. 10.4236/ojs.2016.63043

[4] Kuupiel D Vezi P Bawontuo V : Tuberculosis active case-finding interventions and approaches for prisoners in sub-Saharan Africa: a systematic scoping review. *BMC Infect Dis.* 2020;20(1):1–14. 10.1186/s12879-020-05283-1 PMC7405346 32758165

[5] Otiende V Achia T Mwambi H : Bayesian modeling of spatiotemporal patterns of TB-HIV co-infection risk in Kenya. *BMC Infect Dis.* 2019;19(1):1–13. 10.1186/s12879-019-4540-z 31660883 PMC6819548

[6] Zumla A Petersen E Nyirenda T : Tackling the tuberculosis epidemic in sub-Saharan Africa--unique opportunities arising from the second European Developing Countries Clinical Trials Partnership (EDCTP) programme 2015-2024. *Int J Infect Dis.* 2015;32:46–49. 10.1016/j.ijid.2014.12.039 25809755

[7] Osei E Oppong S Der J : Trends of tuberculosis case detection, mortality and co-infection with HIV in Ghana: A retrospective cohort study. *PLoS One.* 2020;15(6): e0234878. 10.1371/journal.pone.0234878 32579568 PMC7313972

[8] Osei E Oppong S Adanfo D : Reflecting on tuberculosis case notification and treatment outcomes in the Volta region of Ghana: a retrospective pool analysis of a multicentre cohort from 2013 to 2017. *Glob Heal Res policy.* 2019;4(1):1–13. 10.1186/s41256-019-0128-9 PMC6916450 31890895

[9] Ghana HS : The Health Sector in Ghana: Facts and Figures 2018. *Minist Heal Ghana [Internet].* 2018:1–50. Reference Source

[10] Service GH : *The Health Sector in Ghana Facts and Figures 2018 [Internet].* 2021. Reference Source

[11] World Health Organization : STBI. *Treatment of tuberculosis: guidelines.* World Health Organization;2010. 23741786

[12] Service GH . Ghana Health Service Annual Report.Unpublished;2014.

[13] Ghana : *Tuberculosis profile: Ghana.* 2021.

[14] Aryee G Kwarteng E Essuman R : Estimating the incidence of tuberculosis cases reported at a tertiary hospital in Ghana: a time series model approach. *BMC Public Health.* 2018;18:1292. 10.1186/s12889-018-6221-z 30477460 PMC6258486

[15] Aronis JM Ferraro JP Gesteland PH : A Bayesian approach for detecting a disease that is not being modeled. *PLoS One.* 2020;15(2): e0229658. 10.1371/journal.pone.0229658 32109254 PMC7048291

[16] Fouarge E Monseur A Boulanger B : Hierarchical Bayesian modelling of disease progression to inform clinical trial design in centronuclear myopathy. *Orphanet J Rare Dis.* 2021;16(1):1–11. 10.1186/s13023-020-01663-7 33407688 PMC7789189

[17] Lawson AB Lawson AB : *Statistical methods in spatial epidemiology.* John Wiley;2001.

[18] Lawson A Lee D . Bayesian disease mapping for public health. In: Handbook of statistics. Elsevier;2017. pp.443–481.

[19] Lawson AB : *Bayesian disease mapping: hierarchical modeling in spatial epidemiology.* CRC Press;2018. 10.1201/9781351271769

[20] Blangiardo M Cameletti M Baio G : Spatial and spatio-temporal models with R-INLA. *Spat Spatiotemporal Epidemiol [Internet].* 2013;7:39–55. 10.1016/j.sste.2013.07.003 Reference Source 24377114

[21] Blangiardo M Cameletti M : *Spatial and spatio-temporal Bayesian models with R-INLA.* John Wiley & Sons;2015. 10.1002/9781118950203

[22] Lindgren F Rue H : Bayesian spatial modelling with R-INLA. *J Stat Softw.* 2015;63(19). 10.18637/jss.v063.i19

[23] Schrödle B Held L : A primer on disease mapping and ecological regression using INLA. *Comput Stat.* 2011;26(2):241–258. 10.1007/s00180-010-0208-2

[24] Besag J York J Mollié A : Bayesian image restoration, with two applications in spatial statistics. *Ann Inst Stat Math.* 1991;43(1):1–20. 10.1007/BF00116466

[25] Best N : Bayesian Hierarchical Modelling Using WinBUGS. 2011;

[26] Best N Richardson S Thomson A : A comparison of Bayesian spatial models for disease mapping. *Stat Methods Med Res.* 2005;14(1):35–59. 10.1191/0962280205sm388oa 15690999

[27] Kyung M Ghosh SK : Bayesian inference for directional conditionally autoregressive models. *Bayesian Anal.* 2009;4(4):675–706.

[28] Lawson AB : *Bayesian disease mapping: hierarchical modeling in spatial epidemiology.* CRC Press;2013. 10.1201/b14073

[29] Lawson AB : *Bayesian disease mapping: hierarchical modeling in spatial epidemiology.* Chapman & Hall/CRC;2008. 10.1201/9781584888413

[30] Ntzoufras I : *Bayesian modeling using WinBUGS.* Wiley;2011.

[31] Clayton D Kaldor J : Empirical Bayes estimates of age-standardized relative risks for use in disease mapping. *Biometrics.* 1987;43:671–681. 10.2307/2532003 3663823

[32] Clark TG Bradburn MJ Love SB : Survival analysis part I: basic concepts and first analyses. *Br J Cancer.* 2003;89(2):232–238. 10.1038/sj.bjc.6601118 12865907 PMC2394262

[33] Knorr-Held L : Bayesian modelling of inseparable space-time variation in disease risk. *Stat Med.* 2000;19(1718):2555–2567. 10.1002/1097-0258(20000915/30)19:17/18<2555::AID-SIM587>3.0.CO;2-# 10960871

[34] Bernardinelli L Clayton D Pascutto C : Bayesian analysis of space—time variation in disease risk. *Stat Med.* 1995;14(21–22):2433–2443. 10.1002/sim.4780142112 8711279

[35] Waller LA Carlin BP Xia H : Hierarchical spatio-temporal mapping of disease rates. *J Am Stat Assoc.* 1997;92(438):607–617. 10.1080/01621459.1997.10474012

[36] Robert CP Rousseau J : A special issue on Bayesian inference: challenges, perspectives and prospects. *Philos Trans R Soc A Math Phys Eng Sci [Internet].* 2023;381(2247):20220155. 10.1098/rsta.2022.0155 36970829 PMC10041347

[37] Crupi V Calzavarini F : Critique of pure Bayesian cognitive science: A view from the philosophy of science. *Eur J Philos Sci [Internet].* 2023;13(3):28. 10.1007/s13194-023-00533-w

[38] Hahn U : The Bayesian boom: good thing or bad? *Front Psychol [Internet].* 2014;5:5. 10.3389/fpsyg.2014.00765/abstract 25152738 PMC4126207

[39] Roy V : Convergence Diagnostics for Markov Chain Monte Carlo. *Annu Rev Stat Its Appl [Internet].* 2020;7(1):387–412. 10.1146/annurev-statistics-031219-041300

[40] Rue H Martino S Chopin N : Approximate Bayesian inference for latent Gaussian models by using integrated nested Laplace approximations. *J R Stat Soc Ser b (statistical Methodol).* 2009;71(2):319–392. 10.1111/j.1467-9868.2008.00700.x

[41] Lindgren F Rue H : Bayesian spatial modelling with R-INLA. J Stat Softw. 2015;63(19):1–25. 10.18637/jss.v063.i19

[42] Schrödle B Held L : Spatio-temporal disease mapping using INLA. *Environmetrics.* 2011;22(6):725–734. 10.1002/env.1065

[43] Lawson AB :2008; *Bayesian disease mapping: hierarchical modeling in spatial epidemiology.* New York: Chapman and Hall/CRC. 10.1201/9781584888413

[44] Miller HJ : Tobler’s first law and spatial analysis. *Ann Assoc Am Geogr.* 2004;94(2):284–289. 10.1111/j.1467-8306.2004.09402005.x

[45] Bernardinelli L Clayton D Montomoli C : Bayesian estimates of disease maps: how important are priors? *Stat Med.* 1995;14(21–22):2411–2431. 10.1002/sim.4780142111 8711278

[46] Bernardinelli CD Pascutto C Montomoli C : Bayesian analysis of space—time variation in disease risk. *Stat. Med.* 1995;14(21–22):2433–2443. 10.1002/sim.4780142112 8711279

[47] Knorr-Held L Besag J , others. Modelling risk from a disease in time and space. Stat Med. 1998;17(18):2045–2060. 10.1002/(SICI)1097-0258(19980930)17:18<2045::AID-SIM943>3.0.CO;2-P 9789913

[48] Spiegelhalter DJ Best NG Carlin BP : The deviance information criterion: 12 years on. *J R Stat Soc Ser B Stat Methodol.* 2014;76(3):485–493. 10.1111/rssb.12062

[49] Abdul IW Ankamah S Iddrisu AK : Space-time analysis and mapping of prevalence rate of tuberculosis in Ghana. *Sci African.* 2020;7: e00307. 10.1016/j.sciaf.2020.e00307

[50] Fact Sheet-WHO : WHO, Tuberculosis. *Glob Tuberc Rep End TB Strateg.* 2016:2016.

[51] Said H Kachingwe E Gardee Y : Determining the risk-factors for molecular clustering of drug-resistant tuberculosis in South Africa. *BMC Public Health.* 2023;23:1–12. 10.1186/s12889-023-17234-x 38001453 PMC10668341

[52] Dye C Williams BG : The Population Dynamics and Control of Tuberculosis. *Science.* 2010;328(5980):856–861. 10.1126/science.1185449 20466923

[53] Narasimhan P Wood J Macintyre CR : Risk Factors for Tuberculosis. *Pulm Med.* 2013;2013:1–11. 10.1155/2013/828939 PMC3583136 23476764

[54] Knorr-Held L Raßer G : Bayesian detection of clusters and discontinuities in disease maps. *Biometrics.* 2000;56(1):13–21. 10.1111/j.0006-341X.2000.00013.x 10783772

[55] Awine T Silal SP : Assessing the effectiveness of malaria interventions at the regional level in Ghana using a mathematical modelling Application. *PLOS Glob Public Heal.* 2022;2(12): e0000474. 10.1371/journal.pgph.0000474 36962718 PMC10021332

[56] Yeboah-manu D Asare P Otchere ID : Spatio-Temporal Distribution of Mycobacterium tuberculosis Complex Strains in Ghana. *PLoS One.* 2016;11:1–19. 10.1371/journal.pone.0161892 PMC5001706 27564240

