# Supplementary material for: Spatio-temporal characteristics of Tuberculosis in Ghana
Source: F1000Res. 2024 Jun 21;11:200. Originally published 2022 Feb 16. [Version 4] doi: 10.12688/f1000research.109053.4 (PMC11167329; doi:10.12688/f1000research.109053.4)
Supplement: Peer review history

# Reviewer report: Reviewer response for version 4

**DOI:** 10.5256/f1000research.167925.r287290

**Published:** 2024-06-26

**Authors:** Soedarsono Soedarsono

**Affiliations:** 1 Sub-pulmonology Department of Internal Medicine, Faculty of Medicine, Hang Tuah University, Surabaya, Indonesia

This study successfully fills a gap in the literature by applying a hierarchical Bayesian model to identify Tuberculosis 'hotspots' in Ghana. The authors produced a detailed and visual mapping of TB risk, highlighting the significance of factors such as TB cure rates, community knowledge about TB, and HIV prevalence in influencing TB infection risk across different regions of Ghana. This aspect is one of the highlights of the journal. However, there is important point in this manuscript that need clarification as follows:

- The authors have not provided sufficient details regarding the exact methods or procedures used to ensure that the TB patients included in the study were accurately diagnosed both bacteriologically and/or clinically. Further information about the steps taken to validate these diagnoses would provide greater clarity and confidence in the spatial and spatio-temporal analysis results conducted in this study.

Overall, this manuscript needs **Minor Revisions.**

Is the work clearly and accurately presented and does it cite the current literature?

Yes

If applicable, is the statistical analysis and its interpretation appropriate?

I cannot comment. A qualified statistician is required.

Are all the source data underlying the results available to ensure full reproducibility?

Partly

Is the study design appropriate and is the work technically sound?

Yes

Are the conclusions drawn adequately supported by the results?

Yes

Are sufficient details of methods and analysis provided to allow replication by others?

Partly

Reviewer Expertise:

pulmonary TB

I confirm that I have read this submission and believe that I have an appropriate level of expertise to confirm that it is of an acceptable scientific standard, however I have significant reservations, as outlined above.

# Reviewer report: Reviewer response for version 3

**DOI:** 10.5256/f1000research.162565.r271890

**Published:** 2024-06-11

**Authors:** Md. Saiful Islam

**Affiliations:** 1 University of New South Wales, Sydney, New South Wales, Australia

Please see my specific comments below:

***Abstract:***

*The conclusion in the abstract needs improvement. Based on the findings, there should be a summary sentence in the conclusion.*

***Main Manuscript:***

- ***Background:***

- ‘Mycobacteria’ should be *italicized*.

 - "In 2019, around 10 million people tested positive for TB" should be edited to "In 2019, it was estimated that around 10 million people had TB."

 - HIV should be defined when used for the first time.

 - WHO has been defined multiple times. The author may define 'WHO' once, and then there is no need to define it again.

 - Tuberculosis (TB) has already been defined in the first sentence.

- ***Methods:***

"One significant limitation is the reliance on existing data, which could contain errors if healthcare workers input information incorrectly. Additionally, using secondary data may compromise external validity if biases exist in the population under study. Another potential source of bias is non-random participant selection, which could make the estimates unreliable if the sample doesn’t accurately represent the entire population." This sentence should go under the discussion section as study limitations.

- ***Outcome Variables:***

This section needs to be shortened. The author may describe only the outcome variables here. There is no need to present background information that has already been described under the introduction.

- ***Discussion:***

The author may avoid repeating information in the discussion section. For example, the sentence “The data utilized in our analysis are sourced from the Ghana Health Service and the National Tuberculosis Program. To fit the models, we employed the Integrated Nested Laplace Approximation through the R software” has already been discussed earlier. In the discussion, the author may reflect on the fundamental rationale of the study and how the overall results link to the study objectives. The author may provide the primary conclusion that can be logically and defensibly drawn from the results.

 Paragraph 3 under Discussion:

 The study findings indicate that the TB cure rate is associated with an increase in the risk of TB infection. The idea is not clear here. Do you mean the TB cure rate was associated with high case detection? If yes, mention this explicitly.

Is the work clearly and accurately presented and does it cite the current literature?

Partly

If applicable, is the statistical analysis and its interpretation appropriate?

I cannot comment. A qualified statistician is required.

Are all the source data underlying the results available to ensure full reproducibility?

Yes

Is the study design appropriate and is the work technically sound?

Yes

Are the conclusions drawn adequately supported by the results?

Partly

Are sufficient details of methods and analysis provided to allow replication by others?

Yes

Reviewer Expertise:

Emerging infectious diseases, outbreak, infection prevention and control.

I confirm that I have read this submission and believe that I have an appropriate level of expertise to confirm that it is of an acceptable scientific standard, however I have significant reservations, as outlined above.

# Response

**Published:** 2024-06-13

**Authors:** Abdul-Karim Iddrisu

**Affiliations:** University of Energy and Natural Resources, Ghana

**Abstract:**

***Comment 1. 1:*** *The conclusion in the abstract need’s improvement. Based on the findings, there should be a summary sentence in the conclusion.*

**Response 1.1:** We thank the reviewer for these comments. In response, we have revised the conclusion as “Factors influencing tuberculosis (TB) risk in Ghana are TB cure rate, TB success rate, knowledge about TB, HIV prevalence, literacy rate, and income level. The risk distribution was mainly concentrated in the Southern zone, with clusters of TB risk observed among neighboring regions. To achieve a significant reduction in TB cases, it is essential to allocate resources to TB hotspots regions and also implement measures to control significant predictors of TB infection risk.”

**Main Manuscript:**

**Background:**

***Comment 1.2:*** *Mycobacteria’ should be italicized.*

**Response 1.2**: We have italicized “italicized”.

***Comment 1.3:*** *- "In 2019, around 10 million people tested positive for TB" should be edited to "In 2019, it was estimated that around 10 million people had TB."*

**Response 1.3**: We have revised this text.

***Comment 1.4*** *: HIV should be defined when used for the first time.*

**Response 1.4**: Thank you for these comments. In response we have defined HIV as “Human immunodeficiency virus”

***Comment 1.5*** *: WHO has been defined multiple times. The author may define 'WHO' once, and then there is no need to define it again.*

**Response 1.5**: We have revised the text accordingly.

 Comment 1.6: Tuberculosis (TB) has already been defined in the first sentence.

 Response 1.6: We have the text accordingly.            

**Methods:**

**Comments 1.7: “** *One significant limitation is the reliance on existing data, which could contain errors if healthcare workers input information incorrectly. Additionally, using secondary data may compromise external validity if biases exist in the population under study. Another potential source of bias is non-random participant selection, which could make the estimates unreliable if the sample doesn’t accurately represent the entire population." This sentence should go under the discussion section as study limitations.*

**Response 1.7**: We thank the reviewer for this recommendation. In response, the text has been added to the discussion under limitations section.

**Outcome variables:**

**Comment 1.8:** *This section needs to be shortened. The author may describe only the outcome variables here. There is no need to present background information that has already been described under the introduction.*

**Response 1.8**: We have moved the description of the outcome variable to the results section.

**Discussion:**

**Comment 1.9:** *The author may avoid repeating information in the discussion section. For example, the sentence “The data utilized in our analysis are sourced from the Ghana Health Service and the National Tuberculosis Program. To fit the models, we employed the Integrated Nested Laplace Approximation through the R software” has already been discussed earlier. In the discussion, the author may reflect on the fundamental rationale of the study and how the overall results link to the study objectives. The author may provide the primary conclusion that can be logically and defensibly drawn from the results.*

**Response 1.9:** We thank the reviewer for these insightful and useful comments. In response, it is a standard practice to briefly summarize the entire manuscript (from introduction, objective, methods use) and then discussed the results thoroughly.

**Paragraph 3 under Discussion:**

***Comment 2.1*** *: The study findings indicate that the TB cure rate is associated with an increase in the risk of TB infection. The idea is not clear here. Do you mean the TB cure rate was associated with high case detection? If yes, mention this explicitly.*

**Response 2.1:** Thank you for this important observation. In response, yes, we are referring to TB detection. This text has been revised accordingly.

# Reviewer report: Reviewer response for version 2

**DOI:** 10.5256/f1000research.142548.r237979

**Published:** 2024-02-08

**Authors:** Dr. Victor Abiola Adepoju

**Affiliations:** 1 Department of HIV and Infectious Diseases, Jhpiego (An Affiliate of Johns Hopkins University), Abuja, Nigeria

Abstract

 Clarity and Focus: The abstract states, "The spatio-temporal characteristics of TB in Ghana using Bayesian spatial and spatio-temporal regression models was analysed." However, it doesn't detail the novel aspects of these methods or their specific application to the TB context in Ghana, missing an opportunity to highlight the study's unique contributions.

 Specificity: The abstract mentions "baseline predictors of TB infections" but fails to specify which predictors were most significant. For instance, it could be improved by stating, "Among the baseline predictors, factors such as X, Y, and Z were found to be most significant in influencing the TB risk across the ten regions in Ghana."

 Introduction

 Contextual Depth: The introduction provides a global context of TB but lacks specific details about the TB situation in Ghana, particularly the regions chosen for the study. For instance, "Ghana has been affected by the respiratory disease and currently has challenges in eradicating TB."

 Research Gap: The introduction notes the need for the study but could articulate more precisely the specific knowledge gaps, such as "However, there remains a significant gap in understanding the spatio-temporal dynamics of TB in Ghana, which this study aims to address."

 Methods

 Data Source and Collection: The methods section mentions data obtained from "Ghana National Tuberculosis Programme (NTP)" but doesn't discuss the potential limitations or biases in this data collection process.

 Model Assumptions: The section introduces Bayesian models but doesn't critique the assumptions underpinning these models or discuss their potential impact, such as "The Bayesian models assume X, which could influence the results by Y."

 Analytical Robustness: The manuscript could discuss the robustness of the models against issues like overfitting or multicollinearity, or detail any model validation techniques used, which is currently missing.

 Results

 Presentation and Clarity: Results such as "Hot-spots of TB cases are observed in the Upper East, Upper West..." are presented without sufficient interpretation or visualization for easy understanding.

 Statistical Significance and Practical Significance: While statistical results are presented, the practical implications of these findings, especially in the context of public health planning in Ghana, are not thoroughly discussed.

 Discussion

 Comparative Analysis: The discussion could benefit from a more detailed comparison with existing literature, such as "While studies X and Y observed Z, our findings additionally reveal A, suggesting B."

 Policy Implications: The discussion should connect findings to policy implications more explicitly, such as "The identification of TB hot-spots in regions X, Y, and Z suggests a need for targeted interventions in these areas."

 Limitations

 Data Limitations: The limitations section should address the potential impact of data sources and methodologies on the study's reliability, which is not currently discussed.

 Scope of Inference: The manuscript does not discuss the extent to which these findings can be generalized beyond the studied regions.

 Conclusion

 Synthesis of Findings: The conclusion could provide a more concise synthesis of key findings and their importance in the context of TB control in Ghana.

 Forward-Looking Statements: Suggestions for future research based on these findings, which could further improve TB control efforts in Ghana, are missing.

 General Comments

 Flow and Structure: The paper generally follows a logical flow but could ensure a more seamless transition and build-up from one section to the next.

 Grammar and Punctuation: Ensure thorough proofreading to correct any grammatical or punctuation errors.

 Figures, Tables, and References: Figures and tables, such as "Figure 1. Trend of TB cases detection..." and "Table 2. Summary statistics...", should be clearly labelled and referenced in the text, and the references should be current and relevant to the study's subject matter.

Is the work clearly and accurately presented and does it cite the current literature?

Partly

If applicable, is the statistical analysis and its interpretation appropriate?

Partly

Are all the source data underlying the results available to ensure full reproducibility?

Partly

Is the study design appropriate and is the work technically sound?

Partly

Are the conclusions drawn adequately supported by the results?

Partly

Are sufficient details of methods and analysis provided to allow replication by others?

Partly

Reviewer Expertise:

TB, HIV, COVID-19, MPox, Viral hepatitis, NCD and CDs

I confirm that I have read this submission and believe that I have an appropriate level of expertise to confirm that it is of an acceptable scientific standard, however I have significant reservations, as outlined above.

# Response

**Published:** 2024-02-11

**Authors:** Abdul-Karim Iddrisu

**Affiliations:** University of Energy and Natural Resources, Ghana

We would like to thank the reviewer for the many useful and insightful comments. We have now revised the manuscript substantially. We trust that the revised manuscript now meets the required standards for indexing.

 Please find below our responses to the reviewers’ comments. All changes made due to the reviewers comments are highlighted in red text inside the revised manuscript.

**Abstract:**

**Comment 1.1:** Clarity and Focus: The abstract states, "The spatio-temporal characteristics of TB in Ghana using Bayesian spatial and spatio-temporal regression models was analyzed." However, it doesn't detail the novel aspects of these methods or their specific application to the TB context in Ghana, missing an opportunity to highlight the study's unique contributions.

**Response 1.1: **We thank the reviewer these useful comments. In response, we have revised the abstract to highlight the novel aspects the methods used especially their application to TB context in Ghana as “There is insufficient literature regarding the utilization of Bayesian hierarchical models, specifically within the framework of Integrated Nested Laplace Approximation (INLA), for examining the spatial and spatiotemporal dynamics of tuberculosis risk in Ghana. These methods are known to provide precise and efficient estimates of diseases risk in space and time. This study aimed to address this gap by determining TB hotspots regions in Ghana and associated predictors of TB infection risk using the Bayesian modeling framework within the INLA.”

**Comment 1.2:** Specificity: The abstract mentions "baseline predictors of TB infections" but fails to specify which predictors were most significant. For instance, it could be improved by stating, “Among the baseline predictors, factors such as X, Y, and Z were found to be most significant in influencing the TB risk across the ten regions in Ghana.”

**Response 1.2:** We thank the reviewer for these useful comments and suggestions. In response, we have revised the results in the abstract as “Among the baseline predictors, factors such as TB cure rate, TB success rate, knowledge about TB, awareness of airborne transmission of TB, HIV prevalence, percentage of literacy, and high income were found to be most significant in influencing the TB risk across the ten regions in Ghana.”

**Introduction:**

**Comment 1.1:** Contextual Depth: The introduction provides a global context of TB but lacks specific details about the TB situation in Ghana, particularly the regions chosen for the study. For instance, "Ghana has been affected by the respiratory disease and currently has challenges in eradicating TB."

**Response 1.1:** We thank the reviewer for these comments. In response, we have revised the introduction to take into account tuberculosis situation in Ghana (Page 3, 1 ^st^ paragraph), highlighted in a red text.

**Comment 1.2:** Research Gap: The introduction notes the need for the study but could articulate more precisely the specific knowledge gaps, such as "However, there remains a significant gap in understanding the spatio-temporal dynamics of TB in Ghana, which this study aims to address."

**Response 1.2: **Thank for these comments and a very useful suggestion. In response, we have explicitly stated the research gap in the introduction (Page 4, 1 ^st^ paragraph), highlighted in a red text.

**Methods:**

**Comment 1.1:** Data Source and Collection: The methods section mentions data obtained from "Ghana National Tuberculosis Programme (NTP)" but doesn't discuss the potential limitations or biases in this data collection process.

**Response 1.1: **We thank the reviewer for this suggestion. In response, we have revised the Data section accordingly (Page 5, 1 ^st^ paragraph, Section 2.1), highlighted in a red text.

**Comment 1.2: **Model Assumptions: The section introduces Bayesian models but doesn't critique the assumptions underpinning these models or discuss their potential impact, such as "The Bayesian models assume X, which could influence the results by Y."

**Response 1.2:** Thank you for these useful suggestions. We have revised the methodology to include some criticism about the model assumptions (Page 9, last paragraph), highlighted in a red text.

**Comment 1.3:** Analytical Robustness: The manuscript could discuss the robustness of the models against issues like overfitting or multicollinearity, or detail any model validation techniques used, which is currently missing.

**Response 1.3:** Thank you for these comments. We have revised the methods section accordingly (Page 9, 2 ^nd^ paragraph), highlighted in a red text.

**Results:**

**Comment 1.1:** Presentation and Clarity: Results such as "Hot-spots of TB cases are observed in the Upper East, Upper West..." are presented without sufficient interpretation or visualization for easy understanding.

**Response 1.1: **Thank you for these comments. In thank the reviewer for these comments. In response, we have now provided map of Ghana (in Figure 1) to display regions names on the map for easy visualization and identification of regions exhibiting TB hotspots.

**Comment 1.2:** Statistical Significance and Practical Significance: While statistical results are presented, the practical implications of these findings, especially in the context of public health planning in Ghana, are not thoroughly discussed.

**Response 1.2:** We thank the reviewer for these comments. In response, we have revised the discussion section to reflect the practical implications of the study findings (Page 21-2023, Section 4), highlighted in a red text.

**Discussion:**

**Comment 1.1:** Comparative Analysis: The discussion could benefit from a more detailed comparison with existing literature, such as "While studies X and Y observed Z, our findings additionally reveal A, suggesting B."

**Response 1.1:** Thank you for these comments and suggestions. In response, we have revised the Discussion section accordingly (Page 20-23, Section 4), highlighted in a red text.

**Comment 1.2:** Policy Implications: The discussion should connect findings to policy implications more explicitly, such as "The identification of TB hot-spots in regions X, Y, and Z suggests a need for targeted interventions in these areas."

**Response 1.2: **Thank you for the comments and suggestions. We have included the policy briefs in the Conclusion section (Page 23-24, Section 5).

**Limitations:**

**Comment 1. 1:** Data Limitations: The limitations section should address the potential impact of data sources and methodologies on the study's reliability, which is not currently discussed.

**Response 1.1:** We have discussed the potential impact of data sources on the study’s reliability in the Data section (Page 5, 1 ^st^ paragraph).

**Comment 1.2:** Scope of Inference: The manuscript does not discuss the extent to which these findings can be generalized beyond the studied regions.

**Response 1.2:** We thank the reviewer for this recommendation. In response, we have revised the conclusion section with a recommendation for further studies on TB infection risk to consider application of these methodologies using TB data from all the 16 administrative regions in Ghana. “We also recommend application of these methods to study spatial and spatiotemporal patterns of TB infection risk using TB data on all the 16 administrative regions in Ghana.” (Page 23-24, Section 5).

**Conclusion:**

**Comment 1.1:** Synthesis of Findings: The conclusion could provide a more concise synthesis of key findings and their importance in the context of TB control in Ghana.

**Response 1. 1:** We have provided this information the conclusion (Page 23-24, Section 5), highlighted in a red text.

**Comment 1.2:** Forward-Looking Statements: Suggestions for future research based on these findings, which could further improve TB control efforts in Ghana, are missing.

**Response 1.2: **Suggestions for future research have been stated in the conclusion section (Page 23, Section 5), highlighted in a red text.

**General Comments:**

**Comment 1.1: **Flow and Structure: The paper generally follows a logical flow but could ensure a more seamless transition and build-up from one section to the next.

**Response 1.1:** Thank you for positive comments and this useful suggestion to the authors. We have revised the entire manuscript from abstract to references. This is to ensure flow and easy to comprehend. We are much grateful; review comments are very useful, and we hope that our revised manuscript would meet the required standard for publication.

**Comment 1.2:** Grammar and Punctuation: Ensure thorough proofreading to correct any grammatical or punctuation errors.

**Response 1.2:** Thank for these comments. In response, we have proof-read the manuscript for grammar and spelling mistakes.

**Comment 1.3:** Figures, Tables, and References: Figures and tables, such as "Figure 1. Trend of TB cases detection..." and "Table 2. Summary statistics...", should be clearly labelled and referenced in the text, and the references should be current and relevant to the study's subject matter.

**Response 1.3: **We thank the reviewer for these useful suggestions. In response, we have correctly labeled and referenced all figures and tables in the manuscript. We have highlighted the changes in a red text for easy identification of where changes have been made. We have also revised some of the references with current references and relevant to the study subject matter. However, we have also included some old reference based on which these methods were developed.

# Reviewer report: Reviewer response for version 1

**DOI:** 10.5256/f1000research.120511.r156972

**Published:** 2022-12-21

**Authors:** Dioggban Jakperik

**Affiliations:** 1 Department of Statistics, School of Mathematical Sciences, C. K. Tedam University of Technology and Applied Sciences, Navrongo, Ghana

The study modelled TB incidence in Ghana using Bayesian Spatio-Temporal modelling. This covered the old ten (10) administrative regions of Ghana. Regions with high incidents, regarded as hotspots have been identified. The relevant TB and patient characteristics have been considered. The following were however noted which needs consideration:

1. Grammatical errors need to be addressed
2. Ghana now has 16 regions and has to be clearly stated.
3. TB is caused by *bacillus Mycobacterium tuberculosis* and spreads from person to person by air through coughing, etc.
4. Reformat reference number 2 and similar others with such challenges.
5. TB cases should be captured under source of data instead. How did the authors ensure that their data did not involve repetitions since they picked the data from two institutions who play complementary roles in TB management in Ghana?
6. Further, Figures 1,2,3, and 4 are misplaced. They should come under results or maybe introduction is seen as part of literature review but not part of methods.
7. Line 4 after the posterior distribution …(INLA) package.
8. Under Correlated and Uncorrelated heterogeneity section, the third line after Andrew [], delete “a”.
9. Third paragraph under results, delete “L” and all similar such cases.

Is the work clearly and accurately presented and does it cite the current literature?

Yes

If applicable, is the statistical analysis and its interpretation appropriate?

Yes

Are all the source data underlying the results available to ensure full reproducibility?

Yes

Is the study design appropriate and is the work technically sound?

Yes

Are the conclusions drawn adequately supported by the results?

Yes

Are sufficient details of methods and analysis provided to allow replication by others?

Yes

Reviewer Expertise:

Survival and Longitudinal Data Analysis, Survey Theory, and Design of Experiments.

I confirm that I have read this submission and believe that I have an appropriate level of expertise to confirm that it is of an acceptable scientific standard.

# Response

**Published:** 2022-12-23

**Authors:** Abdul-Karim Iddrisu

**Affiliations:** University of Energy and Natural Resources, Ghana

***Comment 1:*** *"Grammatical errors need to be addressed*."

**Response 1** *:  *We thank the reviewer for the comment. In response, we have proof read the manuscript for spellings and grammatical errors

***Comment 2: "*** *Ghana* *now has 16 regions and has to be clearly stated."*

**Response 2: **Thank you for these comments. In response, “The data contain information on TB detection from 2009 to 2017, for only the 10 old administrative regions of Ghana since the newly created 6 additional regions have no data” (See Methods Section under TB cases).

***Comment 3:*** *"T* *B is caused by bacillus Mycobacterium tuberculosis and spreads from person to person by air through coughing, etc."*

**Response 3: **We thank the reviewer for this important recommendation/suggestion. In response, “TB is caused by *bacillus Mycobacterium tuberculosis *and spreads from person to person by air through coughing, etc *.* ^2”   ^(Background Section, lines 2-3).

***Comment*** ***4**: "Reformat reference number 2 and similar others with such challenges."*

**Response 4**: Thank you for these comments. In response we have reformatted the reference as “W. H. Organization; Global tuberculosis report 2020: executive summary.” We have also address similar issues in other references.

***Comment*** ***5*** **:** *"TB cases should be captured under source of data instead. How did the authors ensure that their data did not involve repetitions since they picked the data from two institutions who play complementary roles in TB management in Ghana?"*

**Response 5: **Thank you for this insightful and useful comments. In response, we used data from the two institutions however; our main source is Ghana Health Services and some complementary information from the National Tuberculosis Programme.

***Comment*** ***6: **"Further, Figures 1,2,3, and 4 are misplaced. They should come under results or maybe introduction is seen as part of literature review but not part of methods."*

**Response 6: **Thank you for this useful observations. In response, we have placed these figures under the Methods Section because Methods Section presents source of the data, description of the data and the methods.

***Comment*** ***7: **"Line 4 after the posterior distribution … (INLA) package."*

**Response 7: **Thank you for the comment. In response, we have revised the sentence to now read “The models are implemented in R-software via the Integrated Nested Laplace Approach (INLA) package.”

***Comment*** ***8: **"Line Under Correlated and Uncorrelated heterogeneity section, the third line after Andrew [], delete “a”."*

**Response 8: **Thank you for this observation. In response, we have deleted the “a”.

***Comment*** ***9: **"Third paragraph under results, delete “L” and all similar such cases."*

**Response 9: **Thank you for this observation. In response, we have deleted the “L” and have addressed all similar cases.
